# Graphene impregnated electrospun nanofiber sensing materials: a comprehensive overview on bridging laboratory set-up to industry

**DOI:** 10.1186/s40580-020-00237-4

**Published:** 2020-08-10

**Authors:** Adel Mohammed Al-Dhahebi, Subash Chandra Bose Gopinath, Mohamed Shuaib Mohamed Saheed

**Affiliations:** 1 Department of Fundamental & Applied Sciences, Universiti Teknologi PETRONAS, 32610 Seri Iskandar, Perak Darul Ridzuan Malaysia; 2 Centre of Innovative Nanostructure & Nanodevices (COINN), Universiti Teknologi PETRONAS, 32610 Seri Iskandar, Perak Darul Ridzuan Malaysia; 3 School of Bioprocess Engineering, Universiti Malaysia Perlis, 02600 Arau, Perlis Malaysia; 4 Institute of Nano Electronic Engineering, Universiti Malaysia Perlis, 01000 Kangar, Perlis Malaysia; 5 Department of Mechanical Engineering , Universiti Teknologi PETRONAS , 32610, Seri Iskandar, Perak Darul Ridzuan Malaysia

**Keywords:** Electrospinning, Electrospun nanofibers, Nanocomposites, Graphene, Graphene oxide, Reduced graphene oxide, Graphene quantum dots, Electrochemical biosensors

## Abstract

Owing to the unique structural characteristics as well as outstanding physio–chemical and electrical properties, graphene enables significant enhancement with the performance of electrospun nanofibers, leading to the generation of promising applications in electrospun-mediated sensor technologies. Electrospinning is a simple, cost-effective, and versatile technique relying on electrostatic repulsion between the surface charges to continuously synthesize various scalable assemblies from a wide array of raw materials with diameters down to few nanometers. Recently, electrospun nanocomposites have emerged as promising substrates with a great potential for constructing nanoscale biosensors due to their exceptional functional characteristics such as complex pore structures, high surface area, high catalytic and electron transfer, controllable surface conformation and modification, superior electric conductivity and unique mat structure. This review comprehends graphene-based nanomaterials (GNMs) (graphene, graphene oxide (GO), reduced GO and graphene quantum dots) impregnated electrospun polymer composites for the electro-device developments, which bridges the laboratory set-up to the industry. Different techniques in the base polymers (pre-processing methods) and surface modification methods (post-processing methods) to impregnate GNMs within electrospun polymer nanofibers are critically discussed. The performance and the usage as the electrochemical biosensors for the detection of wide range analytes are further elaborated. This overview catches a great interest and inspires various new opportunities across a wide range of disciplines and designs of miniaturized point-of-care devices.

## Introduction

Recently, the demands for highly sensitive, selective, and low detection limit biosensors to detect the low abundance of analyte molecules have increased substantially not only in biomedical applications but also in food industries, agriculture and environmental monitoring [1]. The development of ultrasensitive devices and new detection approaches for the efficient point-of-care testing with low-cost and high accuracy is an urgent need in the healthcare industry. Biosensors have received tremendous attention as an alternative to the conventional analytical methods due to the unparalleled specificity, sensitivity, rapidity of analysis and the ability to provide a long-term monitoring and a wide range of detection capabilities, including glucose, blood oxygen level, antibodies, mycotoxins, heavy metals in drinking water, pesticides, nucleic acid and body motions pesticides [2]. A variety of approaches have been exploited, including electrochemical biosensors [3–5], fluorescent biosensors [6], colorimetric biosensors [7, 8], potentiometric biosensors [9, 10], optical biosensors [11], and Raman spectroscopy-based platforms [12, 13]. Compared with other detection methods, electrochemistry biosensing platforms provide a more facile, cost-effective and a highly sensitive detection method which enables the fast response-recovery times, monitoring different analytes, and a very low detection limit [14–16]. Recent efforts have focused on improving the sensing features of electrochemical biosensors by increasing the specific surface area of the transducers (interacting materials with the target analyte), where the larger the surface area of the sensing materials, the higher their ability to interact with the medium (analytes) [2].

In recent years, nanocomposite transducers comprising nano-sized materials and polymer matrices have captivated immense attention in the field of advanced materials science due to their remarkably improved thermal, chemical and dimensional stabilities, applicability, electrical conductivity, mechanical and functional properties that can be achieved at relatively lower filler loading [17]. The improved properties are mainly attributed to a very high aspect ratio (in the range of 100–1000) of nano sized fillers, yielding light-weight composites with alterable multifunctional properties which makes them potential candidates for several advanced applications including diagnostics and repair human tissues [18, 19], aid in cellular growth and proliferation [18], detection of pathogens and heavy metals and offer unparalleled platforms for electrochemical biosensing. In particular, nanocomposites made of graphene based nanomaterials (GNMs) with polymers and or nanoparticles such as metals, carbon nanotubes (CNTs), quantum dots, etc., could provide abundant opportunities for fabricating novel sensors and biosensors with enhanced performance [17, 20, 21].

GNMs including graphene, graphene oxide (GO), reduced graphene oxide (rGO) and graphene quantum dots (GQD) have attracted extensive interest in research/industrial applications because of their potential and unique properties. GNMs are suitable for fabricating a wide range of novel biosensors with improved functionalities and analytical capacities thus providing fascinating opportunities for point-of-care detection, lab-on-chip devices, wearable and flexible electronics, foodborne detection, and environmental monitoring [2, 22, 23]. The attractiveness of GNMs transducers relies not only on their ability to act as efficient and stabilizing platforms for the biorecognition elements, but also on their large surface area, small size, physio-chemical properties, high reactivity, high catalytic efficiency, strong adsorption ability, controlled morphology and structure, biocompatibility, and electrocatalytic properties [18, 24]. The favourable structural and compositional synergy of GNMs allows them to be excellent electrode materials for fabricating various sensing platforms [1]. Specifically, the integration of GNMs and electrochemical biosensors has created various ingenious biosensing strategies for applications in the areas of food safety and clinical diagnosis [25].

Despite the great potential of GNMs and polymer nanocomposites, conventional nanocomposite methods including solvent processing, in situ polymerization and the allied processing encounter several issues such as the agglomeration and aggregation of graphene in the polymer matrix solution, the reduction of the electrical and mechanical properties of GNMs as a results of the insulating polymer matrix and poor dispersion of GNMs nanofillers. The aggregation of graphene is caused by its strong intermolecular π–π interaction, and van der Waals forces resulting in a poor dispersion in the polymer matrix [26, 27]. To circumvent these obstacles, electrospinning provides a facile and effective way of incorporating GNMs [28, 29] e.g. GO sheets with very high aspect ratios into the polymer solution overcome the problem of agglomeration since the polymer matrix is converted to nanosized fibers instead of continuous sheets, thus facilitating better dispersion of the exfoliated GO [30]. More importantly, properties such as porosity, elasticity, hydrophobicity, mechanical strength, percolation limit and conductivity can also be tuned by controlling the nanofiller size as well as the electrospinning parameters and solution parameters [31]. Apart from this, GNMs can be decorated on the surface of electrospun nanofibers (ESNFs) using post-processing methods enabling the possibility to fabricate multifunctional GNMs nanostructures with novel and/or improved biosensing performance. GNMs-polymer nanocomposites prepared by electrospinning possess both the advantages of polymers such as lightweight, flexibility and moldability, and special functionality of GNMs such as high strength, thermal stability and electrochemical properties [32]. Furthermore, the functionality and the dispersity of GNMs can be further improved by incorporating secondary phases such as precious metals, metal oxides, gold nanoparticles, CNTs, and hydroxyapatite either during electrospinning or in the post-processing methods, e.g. wet chemical treatment [33]. Owning to their remarkable properties, synergy effect, unique structures and the excellent electron and mass transportation, the ESNF-GNMs composites are potential candidates to improve current technology and open the door to fabricate and commercialize extremely miniaturized new generation biosensors and smart wearable electronics for point-of-care detection in biomedicine and healthcare fields [1, 34, 35].

Electrospinning (electrostatic spinning) involves an electrohydrodynamic process, during which a liquid droplet is electrified to generate a jet, followed by stretching and elongation to generate fibers [36]. Electrospinning setup comprises four essential components namely, a spinneret with a metallic needle (a hypodermic needle with blunt tip) and capillary tube, a syringe pump, a high-voltage–power supply, and a grounded (conductive) metal collecting screen (e.g. aluminum alloy) [37]. The procedure of electrospinning can be elucidated based on four main stages which are electrification, jet initiation and extension, bending instability and further elongation, and solidification of the jet into fibers [38]. ESNFs diameter and morphology play an essential role in constructing biosensors and are controlled by the process parameters (applied voltage, receiving distance and feed rate), solution and solvent conditions (viscosity, concentration, conductivity, surface tension, volatility) and ambient conditions (humidity, temperature, pressure) [28]. Electrospinning has been extensively reviewed with respect to its development, principle and fundamentals, and the critical parameters influencing the fiber diameter and morphology in several recent reviews such as [34, 39–45].

Due to the lack of comprehensive reviews on electrospinning design of GNMs for electrochemical biosensors, this overview aims to adequately exploit the role of electrospun GNMs nanocomposites for designing electrochemical biosensors and sensors with high sensitivity, selectivity and with low detection limits. Additionally, impregnating GNMs into ESNFs either during electrospinning process using pre-processing methods or after electrospinning as surface modification and functionalization using post-processing methods are presented. Besides, the properties of electrospun GNMs nanocomposites (electrochemical, mechanical, thermal stability and electrical conductivity) and their role in electrochemical biosensors design are critically addressed. This review covers a range of electrochemical biosensors and sensors are using electrospun GNMs nanocomposites for the detection of various analytes.

## Graphene-based Nanomaterials (GNMs)

Graphene (the first ever reported 2D paper like lightweight material) is a sp^2^ hybridized carbon atoms that are tightly arranged into hexagonal structures to form a 2D monolayer of graphitic structure analogous to a polycyclic aromatic hydrocarbon of quasi infinite size [46]. As a basic building block of other carbon dimensionalities (allotropes), graphene can be wrapped to generate 0D “buckyballs” (e.g. fullerenes), rolled up to form 1D nanotubes, and stacked to produce 3D graphite [47–49]. Since its discovery in 2004 [50], graphene has been recognized as a “wonder material” mainly due to its atomic crystal multifunctionality which combines remarkable properties such as high electron mobilities in room temperature (250,000 cm^2^/V s) at electron densities of 2 × 10^11^ cm^2^ [51, 52], unparalleled thermal conductivity in the order of 5000 W/mK [53], superlative mechanical strength (Young’s modulus of ~ 1 TPa) [54], large surface area (2630 m^2^/g) [55], and electronic properties, making it attractive for several applications including sensors, biosensors, electronic devices, supercapacitors, spintronic, photonics, flexible and next generation electronics, biomedical applications, energy storage and solar cells [46, 56–65].

There are excellent recent reviews on the use of graphene for medicine and biology applications [66], graphene metal nanocomposites for electrochemical biosensing applications [67], graphene nanocomposites for various applications [68], graphene based biosensors for food contaminates detection [69], graphene for biosensors [70–74], electrochemical sensors [75–79] and sensors [80–82] for biomedical and other downstream applications [73, 77, 78, 83–88].

### GNMs fabrication

**Fig. 1:**
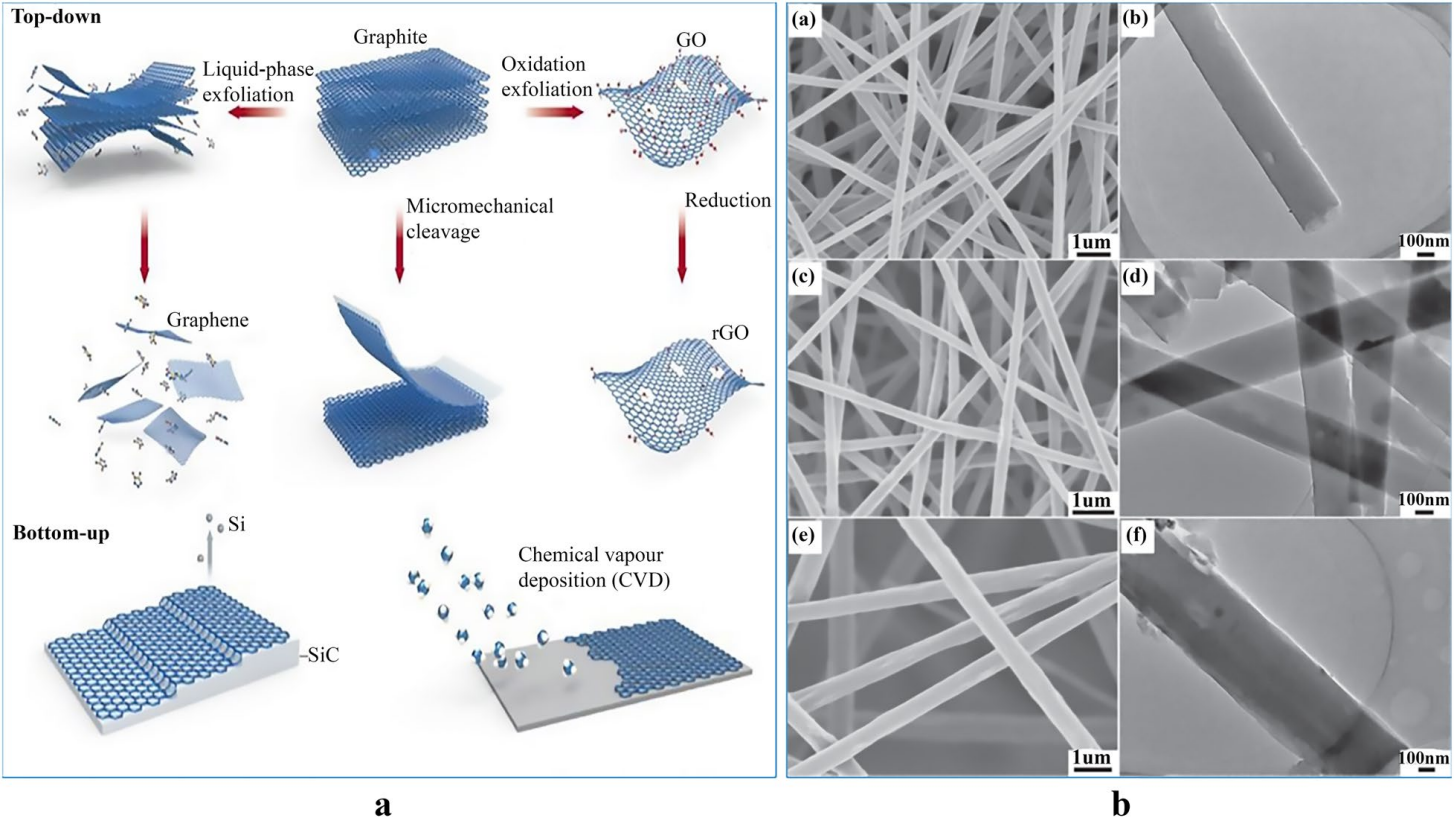
**a** Major fabrication methods of graphene: Top-down and bottom-up fabrication methods. Principal top-down methods include liquid-phase exfoliation and micromechanical cleavage of graphite. An additional method involves the exfoliation of initially oxidized graphite, leading to GO, which is chemically and/or thermally reduced to graphene. The bottom-up fabrication of graphene is usually performed by epitaxial growth on SiC or chemical vapour deposition, typically on Cu using small molecules, such as methane, as precursors. Reproduced with permission from [174] Copyright 2017 Nature Publishing Group. **b** SEM (a, c, e) images and TEM images (b, d, f) of nanofibers (a, b), nanofibers-rGO-5 (c, d), and nanofibers-rGO-10 (e, f) with different magnifications Reproduced with permission from [173] Copyright 2019 Wiley

GNMs include 2D, 3D graphene sheets, GO, rGO, and GQDs can be prepared following two types of fabrication methods: (i) top-down and (ii) bottom-up approaches (Fig. 1a) [89]. The former approach relies on exfoliating stacked layers of graphite by chemical, physical, and thermal treatments to form graphene and it includes micromechanical exfoliation [50], supramolecular assembly [90], conducting polymers [91] and water-soluble polymers [92]. The latter includes chemical vapor deposition (CVD) and chemical synthesis methods [93, 94]. The electrochemistry of graphene and its derivatives depends on the number of defects, functional groups, stacked layers, size of graphene sheets and dopants or impurities present [95–100]. CVD is a vacuum deposition process used to harvest graphene sheets (single or multilayer) with high quality, fine aromatic structures with limited defects, compact constitutes, high reactive surface, electrical conductivity and elasticity making it highly attractive for electrochemical sensing [101] and bioelectrodes to detect molecules and bio-organisms [58, 102–105]. Single-layer graphene (SLG) possess higher electron conductivity at room temperature [250,000 cm^2^/(V s)] [106], thus promoting its applicability for electronics and optoelectronic devices. In principle, the CVD procedure is the shortest and most useful method that allows growing graphene flakes on several substrates (transition metals) such as Ge [107, 108], Ni [109, 110], Cu [111, 112], Rh [113, 114], and etc.

Hummer’s method is a top-down approach to fabricate high-quality and scalable oxidized graphene sheets with different nanosized, good solution process-ability, oxygen content, and sheet layers [115]. GO is an excellent form of graphene [116] having a simultaneous hydrophobic sp^2−^ and sp^3−^ bonded carbon and abundant carboxylic acid groups, epoxide and hydrophilic hydroxyl, especially on the edge and defects of the nanosheet, hence forming a sheet-like amphiphilic colloid [117]. GO, due to its abundant residual sp^2−^ and hydrophilic groups can form stable suspension in aqueous and several polar solvents and form π–π interactions with aromatic molecules [118]. Furthermore, the polar chemical groups, carboxyl acid, epoxide, and hydroxyl on the basal plane allow GO to undergo weak interactions for example strong electrostatic interactions or hydrogen bonding and metal ion complexes which also provide abundant chemically reactive groups for surface grafting/anchoring of polymers and or nanoparticles [2]. The oxidized functional groups of GO improve its dispersion in polymer matrices and minimize the aggregation and phase separation. GO, due to its amphiphilic sheet-like characteristics acts as a surfactant reagent to react with other nanomaterials [117]. RGO, can be obtained by chemical or physical reduction of GO by thermal, chemical, and irradiation methods which are cost-effective approaches to fabricate graphene sheets with a good electrical conductivity. Compared to graphene and GO, rGO has more balanced physical and chemical properties regarding surface chemical groups, electrical, mechanical, solvent dispersibility, optical, and thermal performances [118]. Due to these properties, rGO nanosheets are potential candidates for the next-generation electronics, sensors and transistors. GQDs are nanometer-sized single layer-fragments (their sizes are less than 20 nm in diameter) of graphene and GO, which are typically synthesized via a top-down approach through “cutting” of graphene or GO nanosheets [119]. GQDs exhibit several remarkable physical properties such as the edge defects induced luminescence and the quantum confinement, making GQDs suitable for interesting applications including cell imaging, bioelectrodes and molecular recognition [120–122].

## Electrospun nanofibers containing GNMs

Research pertaining to electrospinning has gained significant traction in recent years, as it provides a versatile and viable tools for generating various matrices in a continuous process and with uniform pore sizes, where the fiber diameters are adjusted from nanometers to sub-microns [40, 123, 124]. ESNFs with diameters lower than 1 nm (subnanometers) have also been recently reported [125, 126]. Although, there are several analogous nanofiber production methods such as nanolithography, self-assembly, melt fibrillation, drawing and template synthesis, electrospinning combines simplicity, low cost and versatility with superior capabilities to manufacture high quality nanofibers with diverse and controlled morphologies and complex nanofibrous assemblies [127–129]. Electrospinning has been successfully applied to produce nanofibers from a wide range of materials, including organic and inorganic polymers, ceramics, metals, graphene, carbon nanotubes, small molecules, and their combinations as well as bacteria, viruses, biomolecules [40, 130, 131]. The incorporation of GNMs into ESNFs enables significant enhancement towards biosensing capability either by improving the response characteristic of the transducer or acting as the immobilisation matrix for a bioreceptor [132]. GNMs can be incorporated into the ESNFs using two main strategies: (i) pre-processing methods (direct blending and in situ synthesis) and (ii) post-processing methods (e.g. physical dip-coating, ultrasonication, plasma treatment, wet chemical method and radiation treatment [68, 133].

### Electrospinning design of GNMs NF composites using pre-processing methods

Introducing GNMs into the polymer solution matrices for electrospinning is a simple and effective method to fabricate electrospun composites for various advanced applications such as sensing and biosensing [28]. In principle, the pre-processing methods consider the size distribution and interface interactions during the encapsulation of GNMs within the polymer nanofibers. In this case, the GNMs should be more stable to ensure the long-term storage stability and excellent reusability of the GNMs ESNFs composite biosensors. In GNMs ESNFs prepared by the pre-processing methods for electrochemical biosensing applications, the GNMs act as the electron transfer platform while the polymers act as a selective adsorptive for bio-tests thus both GNMs and polymers work as a device for electrochemical biosensor electrode. GNMs ESNFs act as a bridge between the test biomolecules and the signal transduction system and thus plays a critical role in both sensor and conductor parts of electrochemical biosensors. High dispersion and even distribution of GNMs within the polymer matrices enable the fabrication of nanofiber composites with highly functional nanofiber composites, novel hierarchical architectures, high specific surface area and tuned porosity, excellent chemical, thermal, electrical and electrochemical properties offering unparalleled performance for point-of-care detection and lab-on-chip devices [132]. There are two effective strategies to ensure uniform distribution of GNMs into polymer nanofibers; the direct blending or mixing of GNMs with polymer matrix before electrospinning and in situ synthesis during electrospinning.

#### Direct blending of GNMs in polymer nanofibers

Blending of GNMs into polymer matrix solution is the basic and straightforward way to fabricate GNMs NFs composites. In this strategy, the direct doping of GNMs into polymer matrix may decrease the surface energy of GNMs which in turn tends to cause local cross-linking between GNMs and polymers. In the case of electrochemical biosensors, the even distribution and dispersion of GNMs within the polymer solution matrices is an essential attribute for improving the linear detection range, sensitivity and limit of detection. Therefore, other ways to improve the dispersity and homogeneity of GNMs within the polymer solution matrices should be investigated.

#### Dispersing GNMs using external forces

One of the main challenges in fabricating nanofibers GNMs is the fact that they have high specific surface area and free energy and tend to agglomerate and/or aggregate which compromise their final performances for biosensor applications [20]. The agglomeration of GNMs may be ascribed to their short-range interactions with the polymeric molecules and the overlapping of interfacial layers of neighbouring graphene nanofillers or polymers. Therefore, if GNMs are not well dispersed and distributed into the polymer matrices at a nanoscale level, the weak molecular interactions take place and the inhomogeneous dispersion may complicate the electrospinnability of solutions, thus reducing the graphene loading capacity and influencing the overall material properties. To overcome these issues, treating the solution with an external force to aid dispersity of GNMs such as manifold repetition of blending and violent stirring, ultrasonic dispersion methods (ultrasonication bath and ultrasonication probe) or by modifying the surface of graphene materials with active surface agents (adding additive to promote the dispersity of GNMs). Adding additives allows mitigating the huge gap in surface energy between the GNMs and the polymer matrices to obtain a better solvability and suitable nano-scaled distribution thus improving their spinnability. Several spacers have been introduced into GNMs to improve the dispersity and to enhance the specific surface area to provide extra adsorption sites for bio and sensing molecules such as metals and metal oxide nanoparticles [134, 135], organic moieties [136, 137], and polymers [134, 135]. Functionalization of graphene using chemical, electrochemical and sonochemical methods improved its dispersion within polymer matrices, for example functionalized graphene such as GO enhances its dispersion in various polymer matrices due to the interfacial interactions between the functionalized graphene and the polymer [138, 139]. Several studies have used external forces and/or adding additives to improve the dispersity and distribution of GNMs in polymer matrix as reported in Table 1.

**Table 1:** Summary of recent significant works on electrospinning design of GNMs with polymer matrices using pre-processing methods

GNMs	Polymer	Solvent	Additives	Dispersion method/external force	ES parameters: (distance; voltage; federate)	Refs.
GO	PVDF	DMF: acetone 4:1 wt/wt%	–	Hydrophobic modification of GO with subsequent sonication and stirring	(27.7 cm; 24.1 kV; 1.23 mL/h)	[140]
rGO	PANCMA	DMF	TiO_2_	Ultrasonication and microwave heating	(30 cm; 14 kV; 0.02 mL/h)	[141]
GO	Poly (lactic acid) (PLA)/poly(butylene carbonate)	DMF solvent	PBC	Stirring	18 kV	[142]
GO	PCL	DMF: DCM 1:1	–	Stirring	14 cm; 18 kV; 10 mL/h	[143]
rGO	poly (ester amide) (PEA)			Ultrasonication bath	(12 cm; 20 kV; 0.1 mL/h)	[144]
GR	PLA	DCM: TFA 2:1 v/v	–	Ultrasonication	(15 cm; 10–20 kV, 2 mL/h)	[33]
GR	PU	THF: DMAC 3:2 w/v	–	Ultrasonication	(10 cm; 15 kV; 1 mL/h)	[145]
GO	PAN	DMF	–	Probe and bath sonication and stirring	(15 cm; 18 kV; 0.2 mL/h)	[146]
Gr	66nylon	TFA: acetone 1:1 v/v	–	Bath and tip sonication	(15–20 cm; 15–20 kV; 0.17 to 0.5 mL/h)	[147]
Gr	Polycaprolactone	DMF	–	Stirring	(10 cm; 10–14 kV; 0.4–0.5 mL/h)	[148]
GO	PLGA	1,1,1,3,3,3-Hexafluoroisopropanol (HFIP)	–	Stirring	(10 cm; 40 kV; 0.07–0.1 mL/min)	[149]
GO/MWCNT	PEO	DMF		Sonication and vigorous stirring	(15 cm; 18.4 kV; 0.5 mL/h)	[150]
GR	Polyamide 66	Formic acid	–	Stirring	(15 cm; 20 kV;0.25 mL/h)	[151]
GO	PVDF	DMF	–	Sonication and stirring	(15 cm; 18 kV; 1 mL/h)	[152]
GO-ZnO	Gum arabic (GA) and PVA		–	Stirring and heating	(130 mm; 0–50 kV)	[130]
GO	Polyurethane (PU)	DMF	Ag	Stirring and heating	(18 cm; 18 kV;1 mL/h)	[153]
GO	poly(Acrylonitrile-co-maleic acid	DMF	–	Microwave heating and ultrasonication	(12; 25 kV; 0.03 mL/h)	[154]
Graphene Nano sheets	poly (Trimethylene terephthalate)	TFA	–	Stirring	(14 cm;	[155]
GO	CA	DMF: acetone 2:3 wt/wt%	–	Sonication and heating and stirring	(15 cm; 27 kV; 0.13 mL/h)	[156]
GO	PLA	DMF	–	Stirring	(6 cm; 20 kV; 1 mL/h)	[157]
rGO	Polystyrene (PS)	(DMF: THF) 1:1	–	Magnetic stirrer	22 kV	[158]
GQD	PAN	DMF	–	DMF Magnetic stirring	240 cm; 15 kV; 0.63 mL/h	[159]
GO	CA	Acetone/DMAc (w/w 2:1)	–	Stirring	(8–10 cm; 20–25 kV; 1.5 mL/h)	[160]
Fluorine-doped GO, GO, and GOCOOH	PVDF	DMAC: acetone (v/v 4:6)	1 g of selectfluor and 0.1 g silver nitrate	Stirring	(12 cm; 25 kV; 0.5 mL/h)	[161]
rGO	PVP/Chitosan	Acetic acid: water 9:1 (w/v)	–	Stirring	(6 cm; 22 kV; 0.5 mL/h)	[162]
rGO	PMMA/PANI	DMF		Stirring and sonication	(15 cm;18–20 kV; 0.3 mL/h)	[163]
GO	PLA/PCL	CF: DMF (v/v = 4/1)	–	Magnetic stirring and sonication	(20 cm; 20 kV)	[164]
GR	PVDF			Sonication and stirring	(17 cm; 20 kV; 1 mL/h)	[165]
GR	PCL	Acetic acid	Gelatin	Sonication and stirring	(15 cm, 10–20 kV; 0.2–1.8 mL/h)	[166]
Gr and GO	PVDF			Ultrasonication probe (100 W, 40 kHz, 15 mints)	(100 mm; 16 kV; 2 mL/h)	[167]

#### In-situ synthesis of GNMs in polymer nanofibers

Similar to blending, the in situ synthesis is an effective strategy to disperse GNMs into the polymer solution to form GNMs NF composites using several methods such as hydrothermal reaction, sol–gel synthesis, oxidation–reduction reaction and hydrolysis. In this strategy, GNMs dispersity in the polymer matrix can be assisted using reactions triggered by light, heat, electrochemistry and reactive additives to uniformly distribute GNMs ions inside the polymer matrix with controlled sizes and uniformity while avoiding the agglomeration of GNMs. Sahatiya and Badhulika [168] reported a facile one step method for in situ synthesis and alignment of a single graphene-doped zinc oxide electrospun nanofiber composite. They optimized the calcination temperature and the time-dependent electrospinning to fabricate aligned graphene-ZnO composite nanofibers across the gold electrode. The reported method is a cost-effective to detect UV and it can be extended to a variety of sensing applications. He et al. [169] reported in situ synthesis, carbonization and electrospinning to fabricate porous graphene-doped copper indium disulfide/carbon (p-GN@CuInS_2_/C) composite nanofibers in which graphene nanosheets anchored with CuInS_2_ nanocrystals of 7–12 nm in diameter were overlapped and embedded in a carbon matrix, aligning along the fiber axial direction. The resultant graphene nanofiber composite exhibited smaller charge-transfer resistance, larger surface area, and excellent electrocatalytic activity than CuInS_2_/C and p-CuInS_2_/C samples.

#### Dispersion of GNMs using electrospinning

Electrospinning applies electrostatic stretching forces to overcome any entanglement and agglomeration of GNMs by increasing their interface contact with the polymer matrix thereby making possible chemical bonds between them. It also provides shear stress transfer mechanics from the polymer matrices to the nanometric of GNMs thus improving the dispersion of GNMs and prevents their aggregation. Additionally, during the electrospinning, the high elongation of the polymer jet improves the orientation and alignment of GNMs along the fiber axis and embeds them in the fiber core thereby achieving highly distributed GNMs-ESNF composites. The content of GNMs influences their dispersion and induces the changes to the solution rheological and physical properties such as electrical conductivity and viscosity and the diameter of the nanofibers. For instance, the increase of GNMs content induces a higher viscosity which in turn results in forming thicker fibers. Meanwhile, the electrical conductivity will rise with the increase of the GNMs content which favours the stretching of thinner fibers [170]. Due to these opposite behaviors, some studies have shown variable fiber diameters as the loading of the nanomaterial is increased [170, 171]. Recently, [172] reported a dual method comprising of electrospinning and electrospraying to overcome the difficulty of blending and dispersing polyacrylonitrile (PAN) and GO in the same solvent. Shan et al. [173] reported the fabrication of a free-standing nitrogen-doped reduced graphene oxide nanofibers using electrospinning technique. The developed nanofibers showed high electronic conductivity and thus has the potential to be used for chemical sensing, separation and drug delivery. Figure 1b) depicts the scanning electron microscope (SEM) results for the developed PAN-GO ESNF mats.

### Electrospinning design of GNMs NF composites using post-processing methods

Although direct blending of GNMs is the simplest and most effective method, one of the critical limitations of blending GNMs into the polymer solution is that as-prepared nanofiber composites may show relatively low-conductivity because the conductivity of GNMs could be warped within the insulating polymers. Alternative approach is to impregnate GNMs onto the surface of ESNFs after electrospinning process using the surface modification methods (post-processing methods). This approach aims at avoiding the problems associated with pre-mixing GNMs into the polymer matrix (e.g. agglomeration and low conductivity) and providing a robust strategy to improve the physiochemical and biological properties of ESNFs. In principle, post-processing methods impregnate or coat GNMs on the surface of the desired ESNFs using chemical or physical strategies to alter the surface of the nanofibers by giving them new features (e.g. surface activation, enhancing surface conductivity) [175]. This induce large number of active sites for further biomolecular immobilization while considering the surface properties of the nanofibers which mainly depends on the chemical composition of the spinning solution and the surface structure of the fibers [176]. This approach is essentially simple and easy to implement and is economically more feasible at an industrial scale than direct mixing of polymers with GNMs. It is worth noting that the arrangement of GNMs should be made to transfer more GNMs to the electrospun polymer nanofiber surface to increase the chance of the interaction between GNMs and bio-analyses which is of great benefit for biosensors [177]. The methods for incorporating electrospun nanofibers with GNMs for sensing applications include physical adsorption and coating, surface graft polymerization, layer-by-layer, plasma modification, chemical doping, heteroatoms doping, wet chemical methods etc. Table 2 summarizes the recent post processing methods used to impregnate ESNFs with GNMs.

**Table 2:** Summary of recent significant works on electrospinning design of GNMs with polymer matrices using post-processing methods

GNMs	ES NFs	Postprocessing method	Mechanism	Potential applications	Refs.
Ag-AQGO	PEO/PVA	Wet chemical route method	The ESNFs were immersed into the as-prepared Ag-AQRGO solution to self-assemble the negatively Ag-AQRGO onto the positively charged NFs in an aqueous solution. The Ag-AQRGO was further washed away with deionized water. After drying in air, the AgNP-3D-AQRGO sensor was obtained	Gas sensors	[32]
PEDOT-CNT/rGO	PVDF-TrFE	Spray coating	PEDOT-CNT/rGO is decorated on ES PVDF-TrFE NFs following these steps: 1. Functionalization of PVDF-TrFE ES NF: using dip coating of ethanol, potassium hydroxide and potassium permanganate and finally hydrogen peroxide. 2. Spraying of the positively charged MCNTs suspension and negatively biased rGO solution on the functionalized PVDF-TrFE ESNF 3. Coating of PEDOT on the substrate to further enhance the electrical conductivity and sensitivity.	Piezo-electric pressure sensor and wearable smart textiles	[33]
rGO	PVP/InCl_3_	Ultrasonic dispersion	The hybrid nanofibers (NFI-rGO) were obtained via ultrasonic dispersion of 2 mg NFI in a rGO aqueous suspension (0.1 mg mL^−1^) for 5 min	Gass sensing in different environments. with 44 ppb detection limit and a response time of 17 s	[34]
rGO	PVA	Cross linking and chemical radiation modification method	The PVA nanofibers were crosslinked (to make them stable and water resistant) with UV-light of 253.7 nm (UV-340 lamp) at 30 W with different duration (15, 30, 45 and 75 min) and then they were kept in both water and PBS solutions to optimize crosslinking duration	Filtration, sensors/biosensors, thin films and packaging	[35]

Among the simplest, fastest and easiest methods to endow electrospun nanofibers with GNMs active sites for target interactions is through the physical dip-coating. This method relies on the interaction between the sensitive probe molecules and the nanofibers which often involves van der Waals forces, hydrophobic forces, electrostatic forces, and hydrogen bonding [178]. However, the efficiency and strength of biomolecular immobilization in this case is relatively weaker [179]. To overcome this limitation, plasma treatment method enables increasing the efficiency of physical absorption onto the hydrophobic nanofibers by creating a more hydrophilic surface thus enhancing biomolecules attachment because of the large availability of carboxyl and hydrophilic surface groups. Layer-by-layer method offer a versatile method to modify the surface of ESNFs by utilizing electrostatic attraction to manipulate the physiochemical, mechanical and biological properties assemble polyelectrolyte multilayers allowing nanoscale control over composition and structure. Chemical doping with atoms is an effective strategy to obtain intrinsic modification of carbon nanomaterials to improve their electrochemical properties [180].

**Fig. 2:**
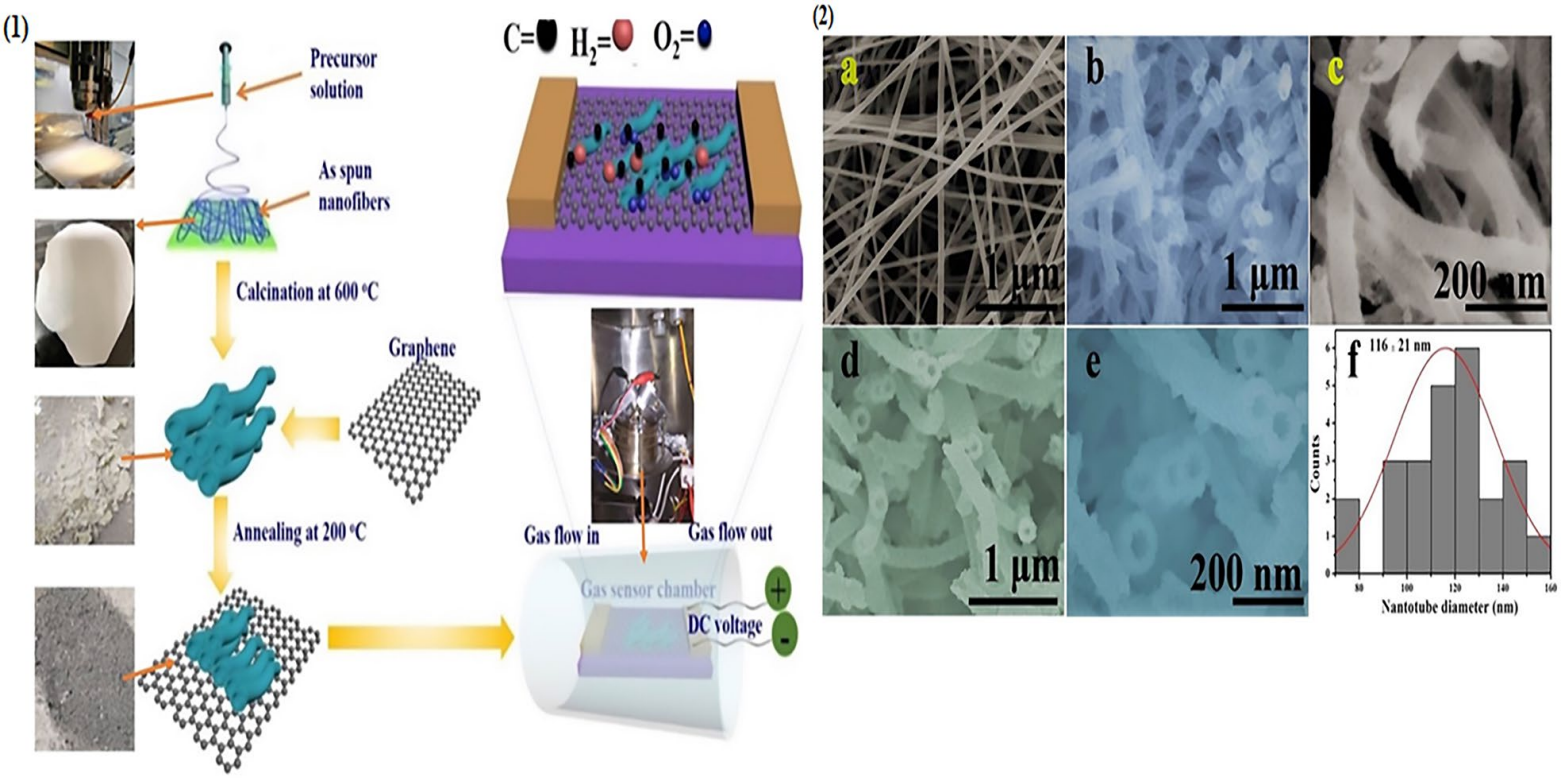
(1): Pristine and GO-SnO_2_ NTs preparation and gas sensor mechanism and (2) SEM images of (**a**) as-prepared Sn + poly (vinyl pyrrolidone) (PVP) nanofibers (**b**, **c**) pristine SnO_2_, and (**d**, **e**) GO incorporate SnO_2_ NTs, (**f**) Histogram of GO-SnO_2_ NT diameters Reproduced with permission from [181] Copyright 2019 Elsevier

Recently, [181] prepared pristine SnO_2_ nanotubes (NTs) by one-step electrospinning and GO was doped into the as-prepared SnO_2_ NTs nanofibers by calcination treatment as shown in Fig. 2(1). First the prepared electrospun SnO_2_ nanotube fibers were annealed at 600 °C for 2 h to remove polymers and the organic residuals and to oxide the inorganic precursors into SnO_2_ nanostructures. Next, 0.03 g of pristine SnO_2_ NTs bundles were dipped into 1 ml of GO (mixed in DI water) solution and dried in the air for several hours. Finally, GO-loaded SnO_2_ were obtained after thermal annealing at 200 °C. SEM images are presented in Fig. 2(2) and the obtained results revealed that the modification of SnO_2_ nanotubes by GO shows the improved sensing properties (e.g. faster response) attributed to the large interfacial interaction between the GO and the SnO_2_ NTs.

Tambakoozadeh et al. [182] utilized in situ polymerization to prepare polyaniline (PANI)/graphene–coated polyamide nanofiber composite for the electrochemical applications. The composite of PANI/GO nanofibers were treated with monohydrate to reduce GO to graphene, and this was followed by the re-oxidation of PANI. The electrical conductivity of the composite PANI/graphene-coated nanofiber was enhanced mainly due to the presence of graphene as well as the increase of aniline concentration in the polymerization process. In terms of the mechanical properties, the presence of GO enhanced the tenacity of the coated nanofibers which is ascribed to the homogenous dispersion of graphene nanosheets and thus the effective load transfer from the matrix to graphene because of their strong interfacial adhension. As for the electrochemical properties, the cyclic voltammetry (CV) curves of the coated nanofibers at a scan of 10 V/s and with a potential window from 0 to 0.9 V (Additional file 1: Figure S1).

Zheng et al. [183] assembled RGO onto the polyurethane (PU) electrospun nanofiber composite assisted by ultrasonication to obtain a polymer core-RGO shell structure. First PU was dissolved in dimethylformamide (DMF) solvent and stirred for 12 h at 60 °C to produce a homogenous solution. The solution was then placed in a syringe and the electrospinning was processed at a flow rate of 1 ml/h, a voltage of 15 kV and the receiving distance was 15 cm. RGO solution was prepared by dispersing RGO in ethanol, water or acetone solvents and ultrasonicated for 0.5 h. The resultant ES PU nanofibers were dipped in the dispersed RGO solution under ultrasonication for different duration from 10 s to 20 min during which RGO nanosheets were gradually assembled on the nanofiber surface to form the core–shell structure. Finally, the RGO decorated composite mat was obtained after washing with ethanol and drying at 60 °C for 12 h. Samani et al. [148] observed an increase in the conductivity and mechanical properties when adding graphene in the polymer matrix for electrospinning. Gozutok et al. [184] dispersed rGO in the poly (vinyl alcohol) (PVA) solutions without using any co-solvent and then electrospinning was used to fabricate nanofiber mats. By adding rGO, the properties of the PVA/rGO NF composite such as the porosity, inter fiber, pore size, and average fiber diameter were relatively improved. It was also observed that, the increase in rGO content improved the mechanical properties, thermal stability and electrical conductivity while the crystal structure of PVA did not change.

## Properties of electrospun GNMs nanocomposites

ESNFs differentiate themselves by their remarkable functional features such as an extremely high surface-area-to volume ratio, ultra-fine diameter, high aspect ratio of length to diameter and molecular orientation along fiber axis, a complex and large porous structure with excellent pore-interconnectivity and tunability, a great mechanical performance, diverse fibrous morphologies, physio–chemical and electrical properties and adjustable structure and diameter [31, 43]. Due to their specialized features, ultrathin diameters and controlled porosity, electrospun nanofiber have demonstrated high potential for a wide spectrum of applications that includes enhancing the performance of analytical devices, biomedical applications, sensor and biosensor technologies [40].

**Fig. 3:**
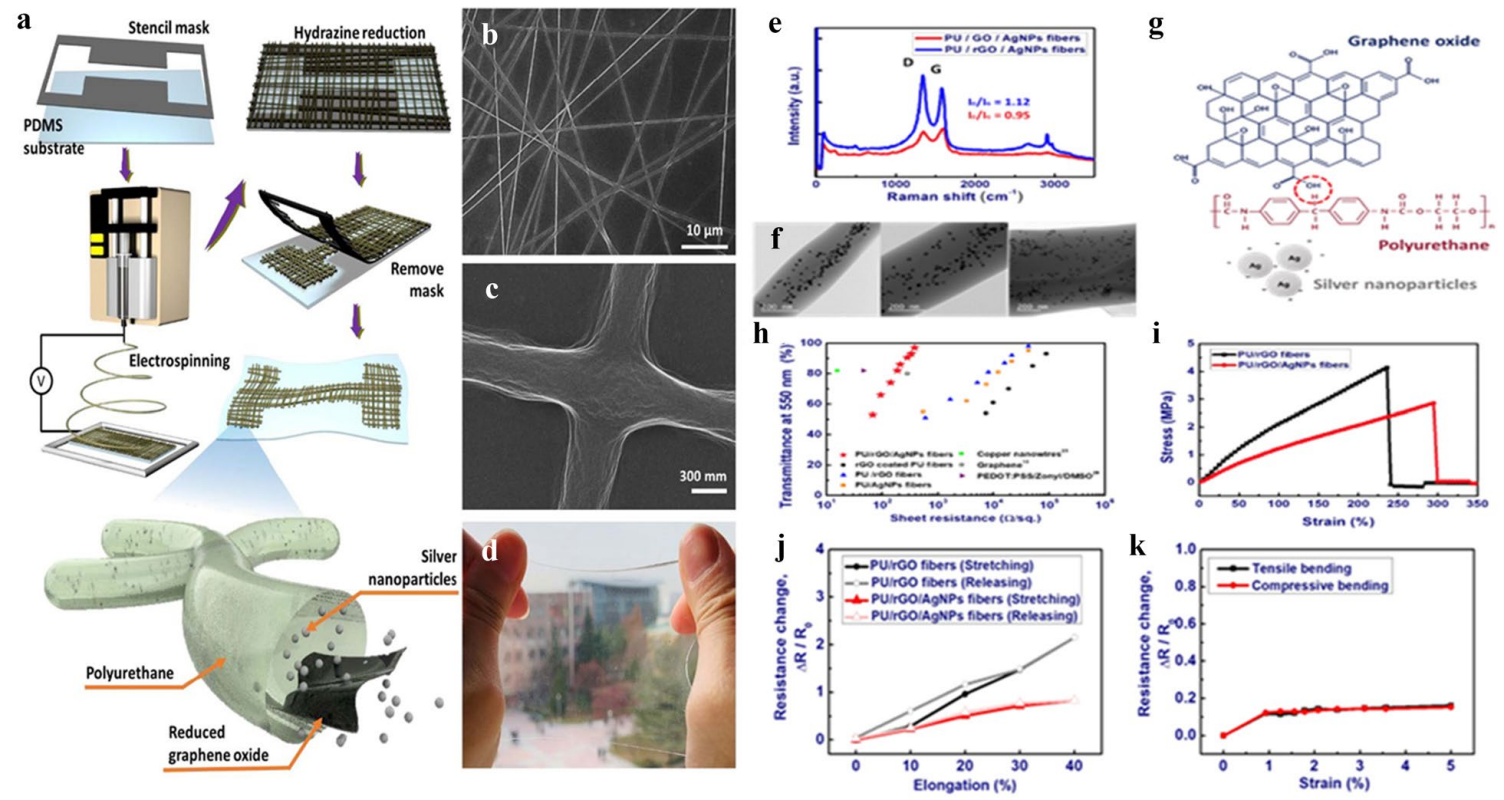
**a** Technological flow chart of the patterned STNNE. **b** FESEM image of the networked nanofibers. **c** FESEM image of the intersections of the nanofibers. **d** Optical photographs of the stretchable and transparent networked nanofibers film. Dispersion of PU/rGO/AgNPs in nanofibers. **e** Raman spectra of PU/GO/AgNPs nanofiber and PU/rGO/AgNPs nanofiber samples with a GO:AgNPs loading ratio of 1:1.25. **f** TEM images of nanofibers with diameters of ~ 290, ~ 484, and ~ 933 nm. **g** Schematics of the functional groups on GO, chemical structure of polyurethane, and negative surface charges of AgNPs. GO nanosheets can be hydrogen-bonded to the PU matrix by the functional moieties of the carboxyl and hydroxyl groups. **h** Optical transmittance-sheet resistance of the networked nanofibers for different types of nanofibers: rGO-coated PU, PU/rGO, PU/AgNPs, and PU/rGO/AgNPs nanofibers with that of copper nanowires, PEDOT: PSS/Zonyl/DMSO and graphene. **i** Stress–strain curves of PU/rGO and PU/rGO/AgNPs nanofibers. Evaluation of STNNEs under stretching conditions. **j** Resistance change (*ΔR/R0*) versus elongation of the PU/rGO and PU/rGO/AgNPs nanofiber electrodes on PDMS substrates. **k** Resistance change (ΔR/R0) versus low strain under tensile and compressive bending of STNNEs Reproduced with permission from [153] Copyright 2019 Royal Society of Chemistry

Impregnating GNMs into ESNFs either during the electrospinning through pre-processing or after electrospinning using post-processing methods impart the nanofibers with remarkable properties and morphological structures, useful for electrochemical sensing and biosensing. In terms of electrochemical properties, the 3D interconnected hierarchical structures of GNMs enable facilitating the diffusion of different types of biomolecules as well as maintain their biocatalytic bioactivity functions thereby improving the sensitive and functionality of biosensors. Owning to their intrinsically high strength derived from the very strong carbon bonds as well as their interactions with the polymer solution matrix and their degree of dispersion, the addition of GNMs can overwhelmingly improve the tensile strength and Young’s modulus of the ESNFs. GNMs are remarkable additives to improve the mechanical and electrical properties of electrospun nanofibers [185]. Dispersion GNMs into polymer matrices have been reported to improve the electrical, mechanical, thermal properties and other properties of polyslfones [186, 187], polyimide [188, 189], polycarbonates [190, 191], polyamides [192, 193], polyethylene terephthalate [192, 193] and polybutylene terephthalate [194, 195]. Gorji et al. [196] reported that the incorporation of GO into electrospun of PU and pH- sensitive dyes contributed to a faster response (7 s) and improved the sensor’s sensitivity to detect pH in chemical vapor solution. Table 3 summarizes the recent studies on impregnating GNMs into ESNFs and the subsequent improved properties. Choi et al. [153] reported a stretchable and transparent nanofiber-networked electrode (STNNE) based on intrinsically stretchable electrospun nanofibers of polyurethane (PU)/reduced graphene oxide (rGO)/silver nanoparticles (AgNPs) (Fig. 3). It was found that, the highly dispersed AgNPs into the PU/rGo nanofibers improved the electrical conductivity, mechanical stretchability. Furthermore, the presence of rGO and the formation of fused intersections between the nanofibers which occurred during the electrospinning process have concert improvements on the electrical stability of the fabricated STNNE. The fabricated STNNE was successfully demonstrated as a stretchable capacitive touch sensor on an elastomeric substrate.

**Table 3:** Summary of studies on the improvement of various properties when adding GNMs in the polymer spinnable solution

GNMs/polymer	Spinning parameters	Improved properties	Potential applications	Remarks	Refs.
Polyacrylonitrile (PAN)/GO	(15 cm; 15 kV; 0.8 mL/h) GO = 0.4%wt	Mechanical strength (by 3–4 times), the thermal stability and hydrophilicity (by 50%)	Water treatment and battery performance	The content of GO influences its dispersion and thus may affect the fiber formation as well as the final performance and properties of ES fibers	[36]
GO/GR/Halloysite NT/PVDF	(10 cm; 16 kV; 2 mL/h)	Piezoelectric and pyroelectric. The thermal stability (by 94%) Young’s modulus increased by 20 times	Wearable electronics and energy harvesting applications from body movements	The content of the nanofillers shows significant effect on piezoelectric responses due to enhancement of electroactive β‐phase	[37]
GO/PEO/PAN	(20 cm; 20 kV; 1 mL/h)	Electrolyte uptake ionic conductivity	The content of GO influences the fiber diameter and ionic conductivity	Homogenous distribution of GO fillers in the polymer matrix causes increase in the electrolyte uptake and electrical conductivity of the nanofibers	[38]
PCL/rGO	(10 cm; 10, 15 kV, 6 mL/h) s	Mechanical behaviour, electrical conductivity and thermal stability	Human tissue repair	The evaluated properties were affected according to the amount of rGO used and the applied voltage	[39]
GO/PET	(12 cm; 10 kV; 0.1 mL/h)	Young modulus by 50% MPa and the electroconductivity	Improve cell attachment and proliferation	The GO, spinning parameters and concentration control the electroconductivity, mechanical properties and the uniformity of NF	[40]
PU-GO-PDA	(20 cm; 20 kV; 0.2 mL/h)	Wettability, water absorption, and both cell attachment and proliferation	Bone regeneration of tissues	PU/GO was prepared by electrospinning and then PDA was coated by immersing PU/GO NF in dopamine hydrochloride solution under constant stirring (1.5 mg/L in 10 mM of Tris buffer pH = 8.5) at room temperature in a dark environment. After 24 h, the scaffold was washed with deionized water three times, and air dried	[41]
GO/(Sulfonated PVA)	(17 cm; 15 to 18 kV; 300 to 800 µL/h)	Thermal and hydrothermal stability, conductivity retention of humidity	Ionic polyelectrolyte membrane	The combination of the sulfonation, the crosslinking, and the addition of GO enhanced the proton conductivity	[42]
PVA/rGO	(6–10 cm; 15–25 kV; 10–20 µL/min)	Tensile strength (~ 5 MPa) and and the elastic modulus (~ 1.5 GPa). Thermal stability and the electrical conductivities	Biosensors, sensors etc.	The increment of rGO (1% wt) improved PVA NF properties due to the strong interfacial interaction between rGO and PVA rGO dispersion in the PVA solution did not alter the crystal structure of PVA	[35]
Ag/rGO/Polyamide (PI)	(20 kV; DMAc: THF = 3:2, wt:wt% Stirring	The λ, Tg and THRI values of the (Ag/rGO)/PI nanocomposites were all increased with increasing the Ag/rGO filler loading	–	The aggregation of rGO can be effectively restricted by introducing Ag nanoparticles –	[43]
PVDF-Pt–Pd/RGO-CeO2	15 cm; 12 kV; 1 mL/h	Increased thermal and catalytic properties	DMFC applications	Novel electrospinning of PVDF-Pt–Pd/RGO-CeO2 nanocomposites	[44]
GO-doped PVDF/CuO/Al	15 cm; 0.07 mm/min	Heat of reaction and reaction efficiency of PVDF/CuO/Al nanocomposites Strong anti-oxidation capability	–	Electrospinning and GO doping can improve the reaction efficiency due to the improvement of microstructure quality and nanocomposites performance	[45]
PU and PU/rGO‐Ag	17 cm; 18 kV; 0.3 mL/h	Tensile strength Electrical conductivity Cardiogenic differentiation Potential Wettability	Cardiac tissue engineering	Adding rGO‐Ag and concentration influence the fiber diameter and the final properties	[46]
MnO2-GO	10 cm; 15 kV; 0.5 mL/h	Electrochemical dielectric behaviors higher charge mobility, diffusivity, and conductivity.	Future energy storage devices	–	[47]
PCL/GO-Gelatin	13 cm; 14 kV; 3 mL/h	Tensile stress and Young’s modulus	Anti-tumor effect of classical therapies	The presence of 1 wt% graphene oxide increased mechanical strength of PCL/Gel	[48]

**Fig. 4:**
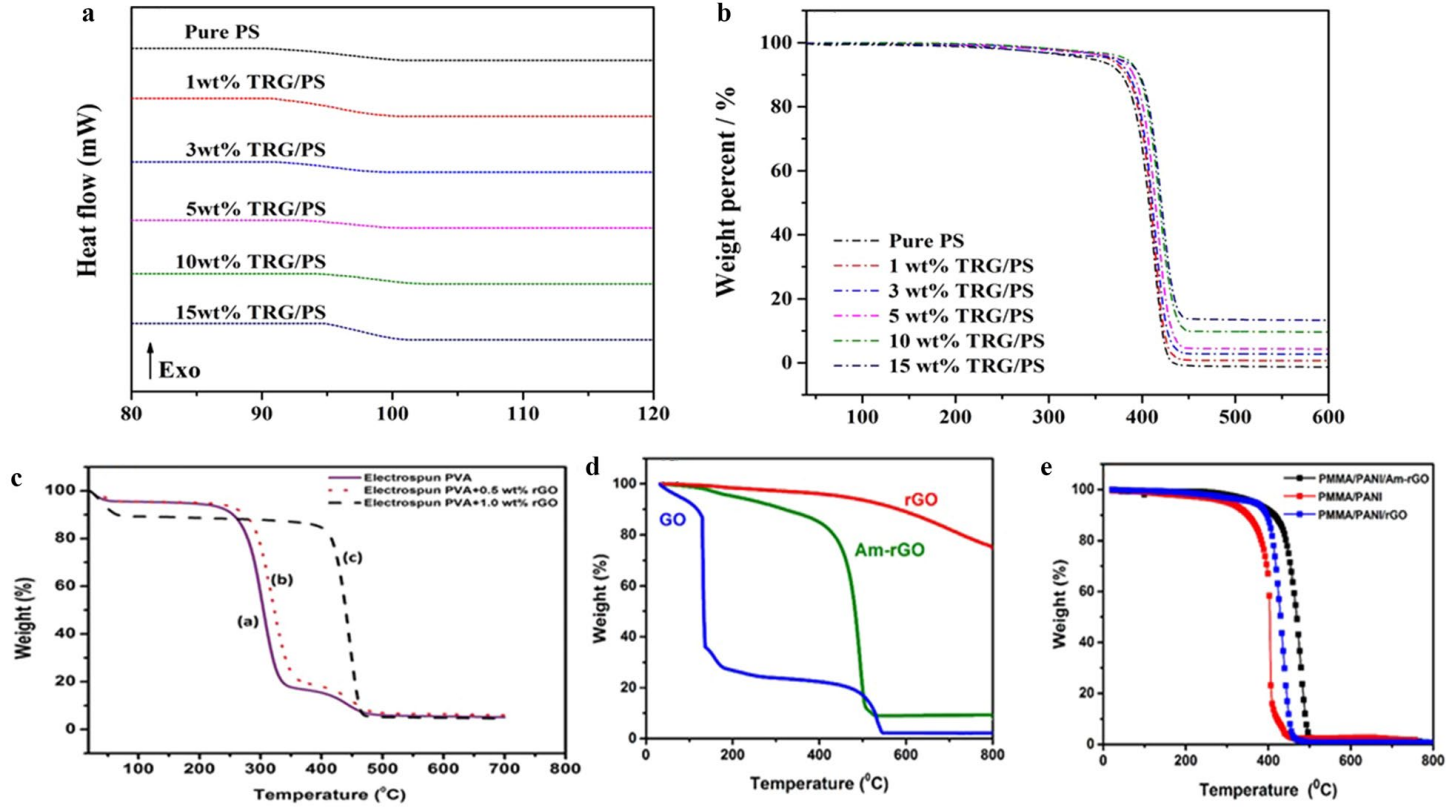
Dimensionally stable anodes (DSC) (**a**) and thermogravimetric analysis (TGA) (**b**) curves of pure PS matrix and the TRG/PS nanocomposites. Reproduced with permission from [158] Copyright 2018 Elsevier. **c** TGA curves of electrospun PVA mats mixed with GO. Reproduced with permission from [184] Copyright 2019 American Scientific Publishers. **d** TGA curves of rGO, rGO and AM-rGO. **e** TGA curves electrospun PMMA/PANI/AM-rGO, PMMA/PANI/rGO and PMMA/PANI nanofibers. As shown in **e**, the thermal degradation temperature of PMMA/PANI/Am-rGO nanofibers increased to ~ 441 °C, a magnitude higher than that of the PMMA/PANI samples at ~ 348 °C. Both **d**, **e** are reproduced with permission from [163] Copyright 2017 MDPI

Ruan et al. [158] reported an increase in the thermal conductivity of polystyrene (PS) as a result of the co-electrospinning of PS with thermally reduced graphene oxide (TRG). More specifically, the addition of 15 wt% TRG could increase the thermally conductive coefficient (λ) value of pure PS from 0.226 to 0.689 W/mK, glass transition coefficient ($a$) value from 0.2157 to 0.6545 mm^2^/s, glass transition temperature (${\text{T}}_{\text{g}}$) value from 90.3 to 95.0 °C and heat-resistance index (${\text{T}}_{\text{HRI}}$) value from 184.2 to 194.3 °C. Gozutok et al. [184] observed that, adding rGO to PVA improved the thermal stability as shown in Fig. 4c. Abdali and Ajji [163] reported that, the thermal stability of PANI improved in the presence of graphene as shown in Fig. 4d, e.

Gebrekrstos et al. [161] reported that the addition of fluoro-doped graphene derivatives (GO, GOF and GOOCH) during electrospinning of polyvinylidene fluoride (PVDF) offered remarkable properties including enhanced electroactive β phase, high energy density and improved piezoelectric coefficient. This drastic enhancement can be ascribed to the increase in the amount of β in PVDF/GO fibers and the charge separation induced by the fluorine which acts as a polarization center. Additional file 1: Figure S2a, b show the piezoelectric response using PFM. Additional file 1: Figure S2c, d show that, adding GO and GOF provided significantly enhanced dielectric constant of PVDF composites due to the fluorine groups that could trap and accumulate large electrons at the interface. Additional file 1: Figure S2e depicts the P-E loops for PVDF and GO, GOF and GOOCH.

## Electrochemical biosensors based electrospun GNMs nanocomposites

Biosensors are analytical devices capable of transferring the response of bio-tests into current signals which comprises two parts, biological detection part and the transduction part. The former is the main part of biosensors which compose of biosensing element (e.g. aptamer, enzyme) that provides selective identification of the bio-tests and converts this detection into processable (current) signals by redox reaction. The latter serves as a platform to transforms the resulting signal from the biomolecule (bioreceptor)-analytes interactions as a current signal to a receiving system for further measurement and quantification. Recently, incorporating GNMs into ESNF to create electrochemical sensors is gaining a wide consideration from researchers mainly because ES GNMs provide a remarkably improved sensitivity and low detection limit caused by their electrochemical probable space, low charge conformation, well-demarcated redox crests, electrocatalytic properties and electron transfer kinetics [197]. Additionally, GNMs possess other excellent characteristics such as high surface area, low-cost, and mass electron transfer ability [155]. In terms of GNMs NFs biosensors, ESNFs serves as the upholder to GNMs as well as the bioreceptors because they possess no reactive ability and thus, do not involve in the detection and transduction parts. Meanwhile, the GNMs act as the detection and transduction parts due to their high adsorption and reactive and abilities for target analytes via chemical bonding or physical adsorption. Highly and uniformly dispersed and distributed GNMs into ESNFs improves the reactivity, speeds up the both adsorption or releases mechanisms and provides large number of GNMs active sites to act as immobilization matrices for bioreceptors (biorecognition elements) in electrochemical biosensors which enhances the electron transfer rate between the biomolecule and the transducer as well as help to preserve their bioactivity on the sensing electrodes [198]. Furthermore, the morphology of ES GNMs NF (porous, core–shell and hollow) contains channels and pores that allows a fluid (e.g. biochemical or chemical species, solvents, gas, etc.) to pass through with minimally reduced mass resistance thereby increasing the analyte diffusion toward the surface of the electrode and provide accurate and ultrasensitive detections [199, 200]. Table 4 shows the summary of ES GNMs and polymer NF composites for sensing applications.

**Table 4:** Summary of studies on ES GNMs and polymer NF composites for biosensing and sensing applications

GNMs/Polymer	Spinning parameters	Limit of detection limits	Biomolecule	Target	References
rGO/PVP/Chi	(12 cm; 22 kV; 0.5 mL/h)	0.15 pmol L^−1^	Laccase enzyme	EE2	[162]
GQDs/PVP	(10 cm; 20 kV; 0.5 mL/l)	12 µM	–	Glucose	[203]
GO/PAN heat treatment > CNT/RGO	15 cm; 15 kV; 1.6 mL/h		–	Electrochemical detection of l-cysteine	[172]
PAN/GO	15 cm; 10 kV; 0.5 mL/h DMF, sonication and stirring		0.25 for lidocaine and 0.5 for prilocaine 2.5 for 2,6-xylidine 1.25 for o-toluidine	Extraction of lidocaine and prilocaine	[204]

Electrospinning is a facile and convenient technique to fabricate nanofibers based biosensors from a wide range of macroporous and mesoporous materials [132]. Electrospinning endow the polymer nanofibers with predictable and controlled pore geometries, desired diameter and thickness, confirmations and chemical functionalities which benefit the fabrication of novel nanostructure materials with biosensing capabilities [199]. Moreover, the opportunity is to modify and functionalize ES NFs on a largescale allows this technique to meet a vast range of sensing requirements over other methods mainly due to the high surface area, high porosity, control of the chemical compositions and the direct electrospinning on a conductive electrode [201]. ESNFs can be functionalized by incorporating GNMs during electrospinning or after electrospinning onto the surface of the as-prepared nanofibers to enhance the essential properties for fabricating electrochemical biosensors (electrical conductivity, electrochemical properties, electron transfer, catalytic reactions). Due to their high specific surface area and high porosity, ESNFs provides immobilizations sites and thus can bind to biorecognition elements through EDC/NHS chemistry enabling biorecognition-analytes interface and enhance the current response for the test biomolecules.

**Fig. 5:**
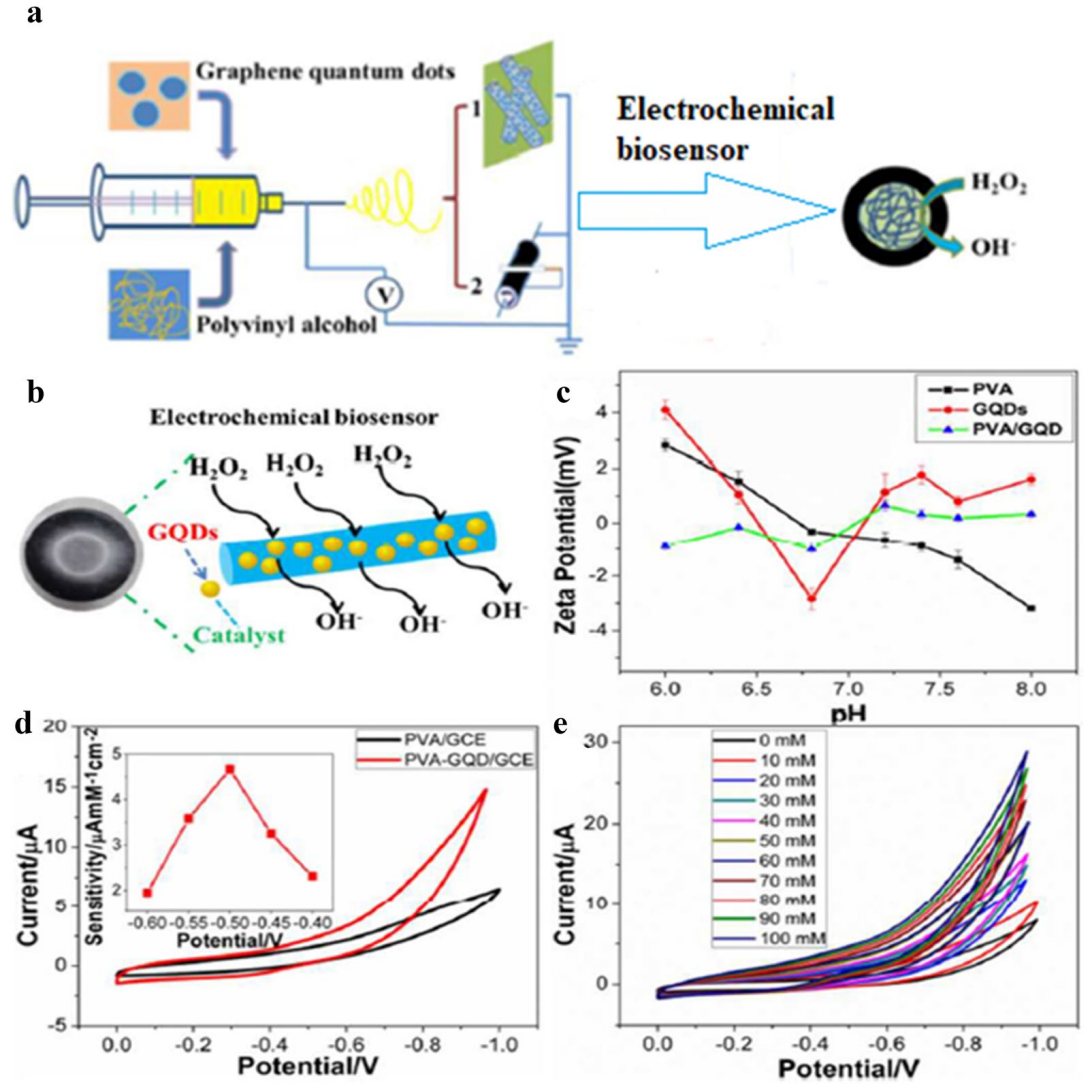
**a** Schematic presentation of electrospinning for producing PVA/GQD onto GCE for electrochemical biosensing and catalyzing of H_2_O_2_, **b** the possible detection mechanism, **c** Zeta potentials of GQDs, PVA, and PVA/GQD nanofibrous membranes at varied pH, **d** CVs of GCEs modified with PVA and PVA/GQD nanofibrous membranes, sensitivity of the biosensor at different potentials (inset), **e** CVs of the PVA/GQD nanofibrous membranes modified GCE 0.1 M PBS with different addition of H_2_O_2_ (Reproduced with permission from [202], Copywrite 2015 Royal Society of Chemistry)

Zhang et al. [202] reported a facile fabrication of a highly sensitive, efficient, stable, and reproducible electrochemical biosensor for H_2_O_2_ detection by electrospinning PVA with GQDs onto glass carbon electrode (GCE) (Fig. 5). GQDs were added into 0.5 g PVA followed by ultrasonication for 2 h and incubation for 10 h. The final concentration of GQDs was 10–50 mM and the obtained homogeneous solution was used for electrospinning PVA/GQDs nanofibrous membrane. The electrospinning parameters were set to 15 kV applied voltage, 12 cm receiving distance, and 0.3–0.5 mL/h flow rate. The ES GQDs electrochemical biosensor showed a linear detection range of 0.1–200 mM and a detection limit of 0.53 μM. It was found that, GQDs can replace the traditional semiconductor QDs and preserve the electrochemical properties of carbon materials.

**Fig. 6:**
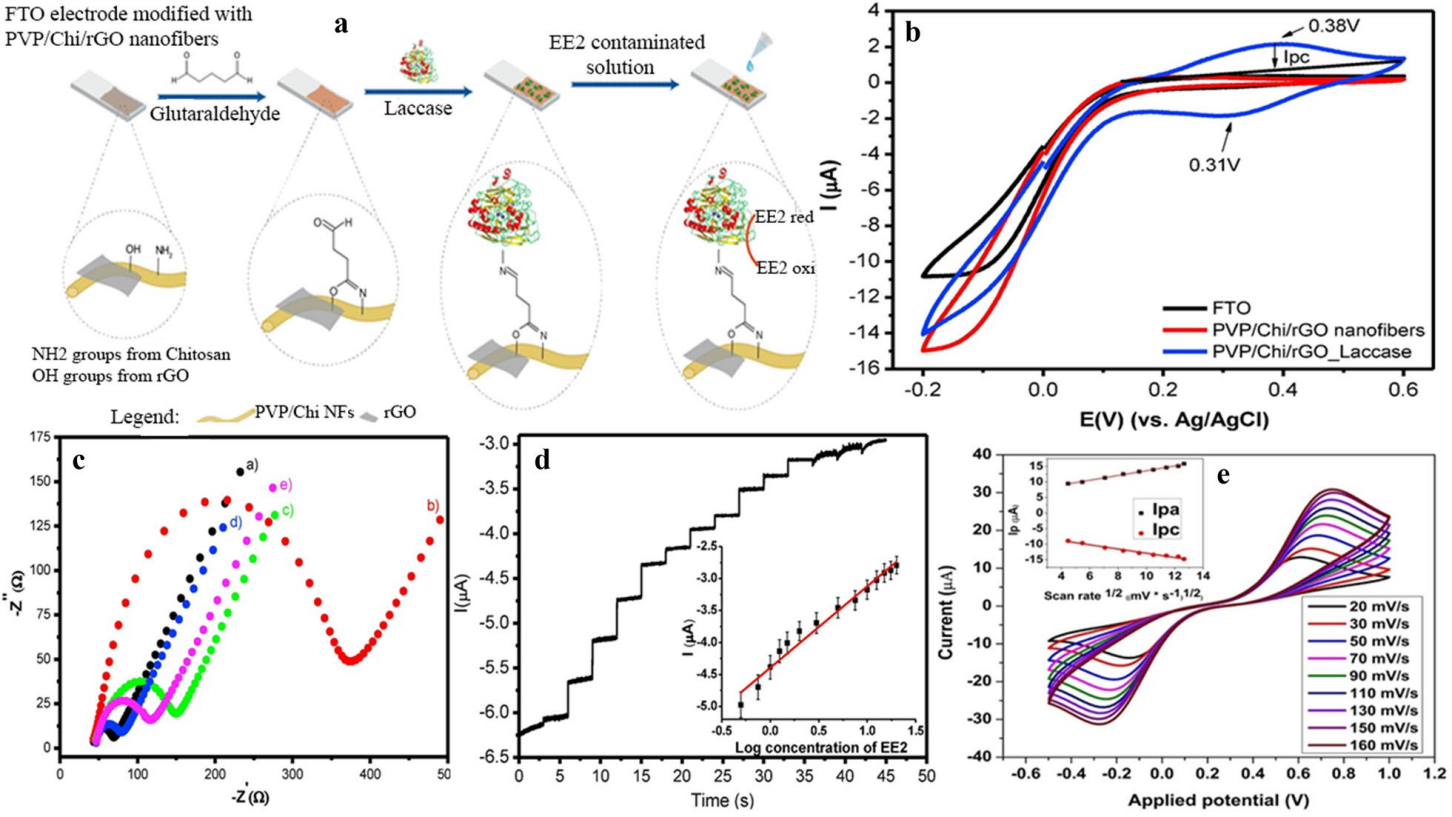
**a** Schematic representation of the fabrication of EE2 electrochemical biosensor. **b** Cyclic voltammetry measurements using a PBS buffer solution (pH 7.4) and scan rate of 100 mV s^−1^ for PTO, PVP/Chi/rGO ES NFs and PVP/Chi/rGO ES NFs coated with Laccase enzyme. **c** Nyquist plots of EIS for (a) FTO, (b) PVP nanofibers, (c) PVP/Chi nanofibers, (d) PVP/Chi/rGO nanofibers and (e) PVP/Chi/rGO nanofibers coated with Laccase in a 5 mmol ${\text{L}}^{ - 1} [{\text{Fe}}\left( {{\text{CN}})_{6} } \right]^{3 - /4 - }$ solution with 0.1 mol L^−1^ KCl. **d** Amperometric response upon successive additions of EE2 ethanol solution recorded at PVP/Chi/rGO_Laccase coated electrode in a phosphate buffer solution pH 7.0 in concentrations ranging from 0.25 to 20 pmol L^−1^ at a fixed potential of − 0.3 V. The inset shows the calibration curve with the respective linear fit. **a**–**d** reproduced from with permission from [162] Copyright 2018 Elsevier. (E) Schematic of cyclic voltammetry shown the electrochemical behaviour of BSA/BH/PNF/GCE in presence of [Fe(CN)_6_]^3−/4−^ at different scan rates (20–160 mV/s). It can be revealed that, the increase in the peak to peak voltage difference is also an indication of the progressive immobilization and the anodic peak shifts towards the higher potential value whereas the cathodic peaks shift towards lower potential value with the increase in the scan rate Reproduced with permission from [129] Copyright 2019 Wiley

Pavinatto et al. [162] proposed a novel ultrasensitive and highly selective electrochemical biosensor based on polyvinylpyrrolidone/chitosan/reduced graphene oxide ES NFs for 17α-Ethinylestradiol (EE2) detection. The spinnable solution was prepared by dispersing 4% w/v of PVP in ethanol and 1.2% w/v chitosan in acetic acid/water (9:1 w/v). Both solutions were mixed and stirred overnight at room temperature before adding 0.035% w/v of rGO which was dissolved in ethanol. The spinning parameters were 22 kV applied voltage, 12 cm receiving distance, and 0.5 mL/h feed rate. The nanofiber composite was deposited on FTO electrodes attached to a metallic collector with a deposition time of 2.5 h. Upon the characterization of the fibers and prior to immobilizing Laccase enzyme, the fabricated PVP/Chi/rGO ESNFs were treated with glutaraldehyde solution and subsequent crosslinking solution was applied to the nanofiber composite to activate the amine (–NH_2_) and the hydroxyl (–OH) groups from chitosan and graphene sheets, respectively. Covalent bonding was utilized to immobilize the Laccase enzyme to the nanofiber composite through NH_2_ groups of Laccase enzyme and the activated groups from the nanofiber composite. The PVP/Chi/rGO/Laccase electrode was used to detect EE2. It was revealed that, the integration of Chi and PVP with rGO increased the charge transfer leading to the excellent electrochemical biosensing properties. Figure 6a reveals the formation of the electrochemical biosensor in terms of coating Laccase enzyme into the FTO/PVP/Chi/rGO nanofiber composite and the CV, electrochemical impedance spectroscopy (EIS) and amperometry measurements are shown in Fig. 6b–d respectively. Recently, Nathani and Sharma [129] demonstrated the use of electrospun mesoporous poly (Styrene-Block-methylmethacrylate) nanofibers (ES PS-b-PMMA NF) to enhance the analytical performance of electrochemical biosensor by exploiting the effect of porosity and surface area on the sensing ability of electropsun nanofibers. EDC-NHS chemistry was chosen to biofunctionalized the PS-b-PMMA NFs and the redox response was utilized to study the presence of the carboxyl group. The fabricated electrochemical porous biosensor showed an increase of the sensitivity by 2.7-fold, a detection range of 10 fM–10 nM and a detection limit of 0.37 fM along with good selectivity. Figure 6e shows the voltammetry results of the developed electrochemical biosensor.

## Future outlook

Electrospinning has become one of the most vital techniques to fabricate the functional nanofiber composites with the desired structure and compositions. However, several challenges hinder the transition of electrospinning method from the laboratory scale to industrial scale production such as spinneret configuration, rheology, solution concentration, electric field intensity and distribution, humidity and temperature, flowrate, receiving distance and collector geometry. These parameters could also influence the reproducibility of ESNFs over time and in different locations. On the other hand, the integration of GNMs and polymer nanofibers using electrospinning has proved to be an excellent strategy to fabricate efficient sensing materials-taking the dual advantages of the wonderful functional properties of GNMs and electrospun polymeric nanofibers. However, to attain high-performance electrochemical biosensors, some challenges should be circumvented such as to increase GNMs contents without agglomeration or aggregation to and to increase the immobilization sites for bio-tests molecules. Additionally, to optimize the synergistic effects between graphene and other nanomaterials as well as to improve the electrocatalytic efficiency for electrochemical sensors are mandatory. There are appropriate modification and fabrication of GNMs and polymer nanofibers for biosensor design via electrospinning which are pre- and post-processing methods. The former involves mixing the polymers with GNMs before electrospinning which is a universal and efficient method to fabricate ES GNMs nanostructures for biosensors with enhanced stability, physical and chemical properties, reusability, and long-term storage stability. The latter involves coating or decorating the GNMs onto the surface of as-prepared nanofibers for immediate interface with biomolecules which in turn leads to the enhanced performance of electrochemical biosensors. The pre-processing methods show more superiorities for biosensing performance; however, they require few harsh conditions like violent stirring, in situ growth of GNMs and/or the use of complicated device such as coaxial electrospinning. Additional challenges of pre-processing methods include the dispersion, alignment and the appropriate loading of GNMs with the polymer matrices. Furthermore, more studies are required to control the synergistic effect of GNMs and their interactions with the polymer matrices during the electrospinning process to ensure uniformity and dispersity of GNMs. The post-processing methods typically have higher efficiency of utilizing GNMs directly for biosensing applications due to the possibility to decorate a large surface area of as-prepared nanofibers with GNMs thus maximizing the potential interface between GNMs and biomolecules to facilitating ultrasensitive detection of bio-tested analytes. The major challenge of post-processing methods lies on their ability to establish accurate interactions between the GNMs and the polymer nanofibers because GNMs cannot easily integrated with the as-prepared nanofibers. Therefore, more studies are required to optimize the coating or to develop novel coating strategy of GNMs onto electrospun nanofibers to increase the interfacial bonding between GNMs and electrospun nanofibers. Recently, [205] reported a facile strategy to realize a strong connection between multi carbon nanotubes (MWCNTs) and poly (vinylidene fluoride-*co*-hexafluoropropylene) ESNFs via thermal-induced welding. Ren et al. [206] reported an effective strategy to improve the structural integrity between CVD graphene and polyacrylonitrile (PAN) ESNFs via annealing process to fabricate a transparent sensor with enhanced conductivity, mechanical strength, sensitivity, stability and a low detection limit.

This review elucidated the recent achievements on electrospun design of functional nanostructures for biosensing applications by exploiting the remarkable properties of GNMs using pre-processing and post processing methods. It can be concluded that, the appropriate modification of GNMs with surface functional groups (e.g. reduction of GO to rGO and or adding additives) improve their dispersion within the polymer matrices thereby enhancing the electrical conductivity, thermal stability, electrochemical and mechanical properties of the electrospun nanostructured composites. Additionally, the modification of electrospun nanofibers as well as optimizing electrospinning design to fabricate porous, core–shell and hollow nanostructures increase the surface area and therefore the immobilization sites for biomolecules increases. This overview highlighted the recent progress on graphene fabrication materials, the remarkable role of GNMs to construct next generation electro-sensing devices and the importance of electrospinning designs of nanostructured composites towards bridging laboratory set-up to the industry.

## Supplementary information

**Additional file 1: Figure S1.** Cyclic voltammetry curves of (**A**) polyaniline (PANI)–coated nanofibers (NP3) and (**B**) PANI/graphene–coated nanofibers (NP3G2). Schematic representation of the charge/discharge curves of (**C**) polyaniline (PANI)–coated nanofibers (NP3) and (**D**) PANI/graphene–coated nanofibers (NP3G2) Ref. [31]. **Figure S2.** Piezoelectric force microscopy (PFM) amplitude vs dc voltage (voltage varying from −12 to 12 V) hysteresis loops for (**a**) PVDF/GOCOOH and (**b**) PVDF/GOF. Frequency dependence of (**c**) dielectric constant and (d) dielectric loss for PVDF/GO, PVDF/GOCOOH, and PVDF/GOF composites. (**e**) schematic representing the P − E loops for PVDF/GO, PVDF/GOCOOH, and PVDF/GOF at room temperature. (**a**–**e**) are obtained from Ref [24] Copy Right ACS.

## Data Availability

All data and material will be made available upon request.

## Abbreviations

CV: Cyclic voltammetry
CVD: Chemical vapor deposition
CNTs: Carbon nanotubes
DMF: Dimethylformamide
EIS: Electrochemical impedance spectroscopy
ESNFs: Electrospun nanofibers
GNMs: Graphene-based nanomaterials
GO: Graphene oxide
rGO: Reduced graphene oxide
GQD: Graphene quantum dots
PAN: Polyacrylonitrile
PANI: Polyaniline
PS: Polystyrene
PU: Polyurethane
PVA: Poly (vinyl alcohol)
PVDF: Polyvinylidene fluoride
PVP: Poly (vinyl pyrrolidone)
SEM: Scanning electron microscope
