# Supplementary material for: Graphene impregnated electrospun nanofiber sensing materials: a comprehensive overview on bridging laboratory set-up to industry
Source: Nano Converg. 2020 Aug 10;7:27. doi: 10.1186/s40580-020-00237-4 (PMC7417471; doi:10.1186/s40580-020-00237-4)
Supplement: Supplementary file 1 — Additional file 1: Figure S1. Cyclic voltammetry curves of (A) polyaniline (PANI)–coated nanofibers (NP3) and (B) PANI/graphene–coated nanofibers (NP3G2). Schematic representation of the charge/discharge curves of (C) polyaniline (PANI)–coated nanofibers (NP3) and (D) PANI/graphene–coated nanofibers (NP3G2) Ref. [31]. Figure S2. Piezoelectric force microscopy (PFM) amplitude vs dc voltage (voltage varying from −12 to 12 V) hysteresis loops for (a) PVDF/GOCOOH and (b) PVDF/GOF. Frequency dependence of (c) dielectric constant and (d) dielectric loss for PVDF/GO, PVDF/GOCOOH, and PVDF/GOF composites. (e) schematic representing the P − E loops for PVDF/GO, PVDF/GOCOOH, and PVDF/GOF at room temperature. (a–e) are obtained from Ref [24] Copy Right ACS. [file 40580_2020_237_MOESM1_ESM.docx]

**Supplementary Information**

Graphene Impregnated Electrospun Nanofiber Sensing Materials: A Comprehensive Overview on Bridging Laboratory Set-up to Industry

Adel Mohammed Al-Dhahebi^1,2^, Subash Chandra Bose Gopinath^3,4^, Mohamed Shuaib Mohamed Saheed^2,5*^

^1^Department of Fundamental & Applied Sciences, Universiti Teknologi PETRONAS, 32610 Seri Iskandar, Perak Darul Ridzuan, Malaysia.

^2^Centre of Innovative Nanostructure & Nanodevices (COINN), Universiti Teknologi PETRONAS, 32610 Seri Iskandar, Perak Darul Ridzuan, Malaysia

^3^School of Bioprocess Engineering, Universiti Malaysia Perlis, 02600 Arau, Perlis, Malaysia.

^4^Institute of Nano Electronic Engineering, Universiti Malaysia Perlis, 01000 Kangar, Perlis, Malaysia.

^5^Department of Mechanical Engineering, Universiti Teknologi PETRONAS, 32610 Seri Iskandar, Perak Darul Ridzuan, Malaysia.

*corresponding author: shuaib.saheed@utp.edu.my

**Table S1.** Summary of recent significant works on electrospinning design of GNMs with polymer matrices using pre-processing methods

| **GNMs** | **Polymer** | **Solvent** | **Additives** | **Dispersion method/ External force** | **ES parameters: (Distance; Voltage; federate)** | **Reference** |
| --- | --- | --- | --- | --- | --- | --- |
| GO | PVDF | DMF: acetone  4:1 wt/wt% | - | ﻿Hydrophobic modification of  GO with subsequent sonication and stirring | (27.7 cm; 24.1 kV; 1.23mL/h) | (H. Li et al., 2020) |
| rGO | ﻿ PANCMA | DMF | ﻿ TiO2 | Ultrasonication and microwave heating | (30 cm; 14 kV; 0.02 mL/h) | (Du et al., 2020) |
| GO | ﻿Poly (lactic acid) (PLA)/poly(butylene carbonate) | DMF solvent | PBC | Stirring | 18 kV | (Gu et al., 2019) |
| GO | PCL | DMF: DCM 1:1 | - | Stirring | 14 cm; 18 kV; 10 mL/h | (Parandeh, Kharaziha, & Karimzadeh, 2019) |
| rGO | ﻿poly (ester amide) (PEA) |  |  | Ultrasonication bath | (12cm; 20kV; 0.1mL/h) | (Stone, Lin, & Mequanint, 2019) |
| GR | PLA | DCM: TFA  2:1 v/v | - | Ultrasonication | (15cm; 10-20kV,2mL/h) | (Darzi, Golestaneh, Kamali, & Karimi, 2019) |
| GR | PU | THF: DMAC  3:2 w/v | - | ultrasonication | (10 cm; 15 kV; 1mL/h) | (Bahrami, Solouk, Mirzadeh, & Seifalian, 2019) |
| GO | PAN | DMF | - | Probe and bath sonication and stirring | (15 cm; 18 kV; 0.2 mL/h) | (Ghaderi, Tavanai, & Bazarganipour, 2019) |
| Gr | 66nylon | TFA: acetone  1:1 v/v | - | Bath and tip sonication | (15-20 cm; 15-20 kV; 0.17 to 0.5 mL/h) | (Maccaferri, Mazzocchetti, Benelli, Zucchelli, & Giorgini, 2019) |
| Gr | Polycaprolactone | DMF | ﻿- | Stirring | (10 cm; 10-14 kV; 0.4-0.5 mL/h) | (Samani, Doostmohammadi, Nilforoushan, & Nazari, 2019) |
| GO | PLGA | ﻿1,1,1,3,3,3-hexafluoroisopropanol (HFIP) | - | Stirring | (10 cm; 40 kV; 0.07-0.1 mL/ min) | (Pan et al., 2019) |
| GO/MWCNT | PEO | DMF |  | Sonication and vigorous stirring | (15 cm; 18.4 kV; 0.5 mL/h) | (Banitaba, Semnani, Heydari‐Soureshjani, Rezaei, & Ensafi, 2019) |
| GR | Polyamide 66 | ﻿formic acid | - | Stirring | (15cm; 20kV;0.25 mL/h) | (Jahan Biglari, Semnani Rahbar, Shabanian, & Khonakdar, 2018) |
| GO | PVDF | DMF | - | Sonication and stirring | (15 cm; 18 kV; 1 mL/h) | (Ren et al., 2019) |
| GO-ZnO | Gum Arabic (GA) and PVA |  | - | Stirring and heating | (130 mm; 0-50 kV) | (Silvestri et al., 2019) |
| GO | Polyurethane (PU) | DMF | Ag | Stirring and heating | (18 cm; 18 kV;1 mL/h) | (Choi et al., 2019) |
| GO | poly(acrylonitrile-co-maleic acid | DMF | - | Microwave heating and ultrasonication | (12; 25 kV; 0.03 mL/h) | (Weng et al., 2019) |
| Graphene Nano sheets | poly (trimethylene terephthalate) | TFA | - | Stirring | (14 cm; | (Huang, Wu, Jeng, & Liang, 2019) |
| GO | CA | DMF: acetone  2:3 wt/wt% | - | Sonication & heating and stirring | (15 cm; 27 kV; 0.13 mL/h) | (Aboamera, Mohamed, Salama,  Osman, & Khattab, 2018) |
| GO | PLA | DMF | - | Stirring | (6 cm; 20 kV; 1 mL/h) | (Davoodi, Mazinani, Sharif, & Ranaei-Siadat, 2018) |
| rGO | Polystyrene (PS) | (DMF: THF) 1:1 | - | Magnetic stirrer | (22 kV; | (Ruan et al., 2018) |
| ﻿GQD | PAN | DMF | - | ﻿  DMF Magnetic stirring | 240 cm; 15 kV; 0.63 mL/h | (Ruiz, Pérez-Marquez, Maudes, Grande, & Murillo, 2018) |
| GO | CA | Acetone/DMAc (w/w 2:1) | - | Stirring | (8-10 cm ;20-25 kV; 1.5 mL/h) | (Javed et al., 2018) |
| Fluorine-doped GO, GO, and GOCOOH | PVDF | DMAC: acetone (v/v 4:6) | ﻿1 g of selectfluor and 0.1 g silver nitrate | Stirring | (12 cm; 25 kV; 0.5 mL/h) | (Gebrekrstos, Madras, & Bose, 2018) |
| rGO | PVP/Chitosan | Acetic acid: water  9:1 (w/v) | - | Stirring | (6 cm; 22 kV; 0.5 mL/h) | (Pavinatto et al., 2018) |
| rGO | PMMA/PANI | DMF |  | Stirring and sonication | (15 cm;18-20 kV; 0.3 mL/h) | (Abdali & Ajji, 2017) |
| GO | PLA/PCL | CF: DMF (v/v=4/1) | - | Magnetic stirring and sonication | (20 cm ;20 kV; | (Wang et al., 2017) |
| GR | PVDF |  |  | Sonication and stirring | (17cm; 20 kV; 1mL/h) | (Abolhasani, Shirvanimoghaddam, & Naebe, 2017) |
| GR | PCL | Acetic acid | gelatin | Sonication and stirring | (15cm, 10-20kV;0.2-1.8mL/h) | (Heidari, Bahrami, & Ranjbar-Mohammadi, 2017) (Heidari et al., 2017) |
| Gr and GO | PVDF |  |  | Ultrasonication probe (100 W, 40kHz, 15 mints) | (100mm; 16kV;2mL/h) | (Abbasipour, Khajavi, Yousefi, Yazdanshenas, & Razaghian, 2017) |


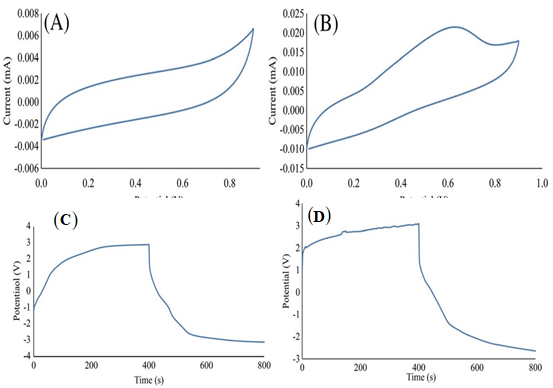


**Figure S1.** Cyclic voltammetry curves of (**A**) polyaniline (PANI)–coated nanofibers (NP3) and (**B**) PANI/graphene–coated nanofibers (NP3G2). Schematic representation of the charge/discharge curves of (**C**) polyaniline (PANI)–coated nanofibers (NP3) and (**D**) PANI/graphene–coated nanofibers (NP3G2). Reproduced with permission from (Tambakoozadeh, Youssefi, & Semnani, 2019) Copyright 2019 Wiley.

**Table S2.** Summary of recent significant works on electrospinning design of GNMs with polymer matrices using post-processing methods

| **GBMs** | **ES NFs** | **Postprocessing method** | **Mechanism** | **Potential Applications** | **Reference** |
| --- | --- | --- | --- | --- | --- |
| Ag-AQGO | PEO/PVA | ﻿Wet chemical route method | The ESNFs were immersed into the as-prepared Ag-AQRGO solution to self- assemble the negatively Ag-AQRGO onto the positively charged NFs in an aqueous solution. The Ag-AQRGO was further washed away with deionized water. After drying in air, the AgNP-3D-AQRGO sensor was obtained. | Gas sensors | (F. Li et al., 2019) |
| PEDOT-CNT/rGO | PVDF-TrFE | ﻿Spray coating | PEDOT-CNT/rGO is decorated on ES PVDF-TrFE NFs following these steps:  1. Functionalization of PVDF-TrFE ES NF: using dip coating of ethanol, potassium hydroxide and potassium permanganate and finally hydrogen peroxide.  2. ﻿Spraying of the positively charged MCNTs suspension and negatively biased rGO solution on the functionalized PVDF-TrFE ESNF  3. Coating of PEDOT on the substrate to further enhance the electrical conductivity and sensitivity. | Piezo-electric pressure sensor and wearable smart textiles | (Ahmed et al., 2019) |
| rGO | PVP/InCl_3_ | Ultrasonic dispersion | The hybrid nanofibers (NFI-rGO) were obtained via ultrasonic dispersion of 2 mg NFI in a rGO aqueous suspension (0.1 mg mL^−1^) for 5 min | Gass sensing in different environments. with 44 ppb detection limit and a response time of 17 s | (Andre, Mercante, Facure, Mattoso, & Correa, 2019) |
| rGO | PVA | Cross linking and chemical radiation modification method | The PVA nanofibers were crosslinked (to make them stable and water resistant) with UV-light of 253.7 nm (UV-340 lamp) at 30 W with different duration (15, 30, 45 and 75 min) and then they were kept in both water and PBS solutions to optimize crosslinking duration | filtration, sensors/biosensors, thin films and packaging. | (Gozutok, Sadhu, & Sasmazel, 2019) |


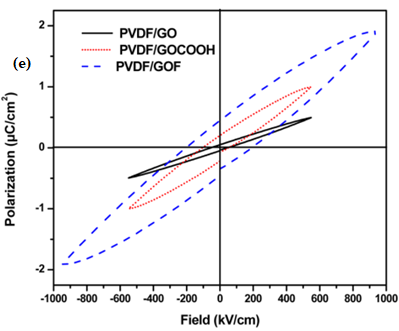

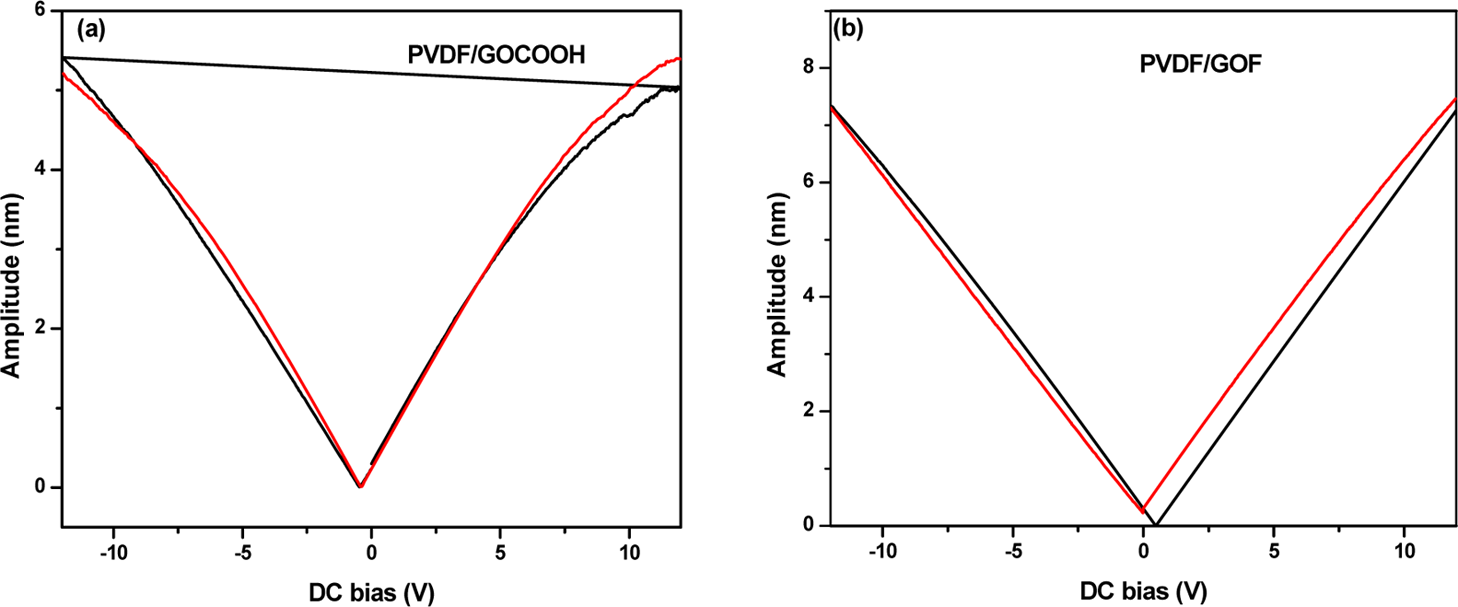

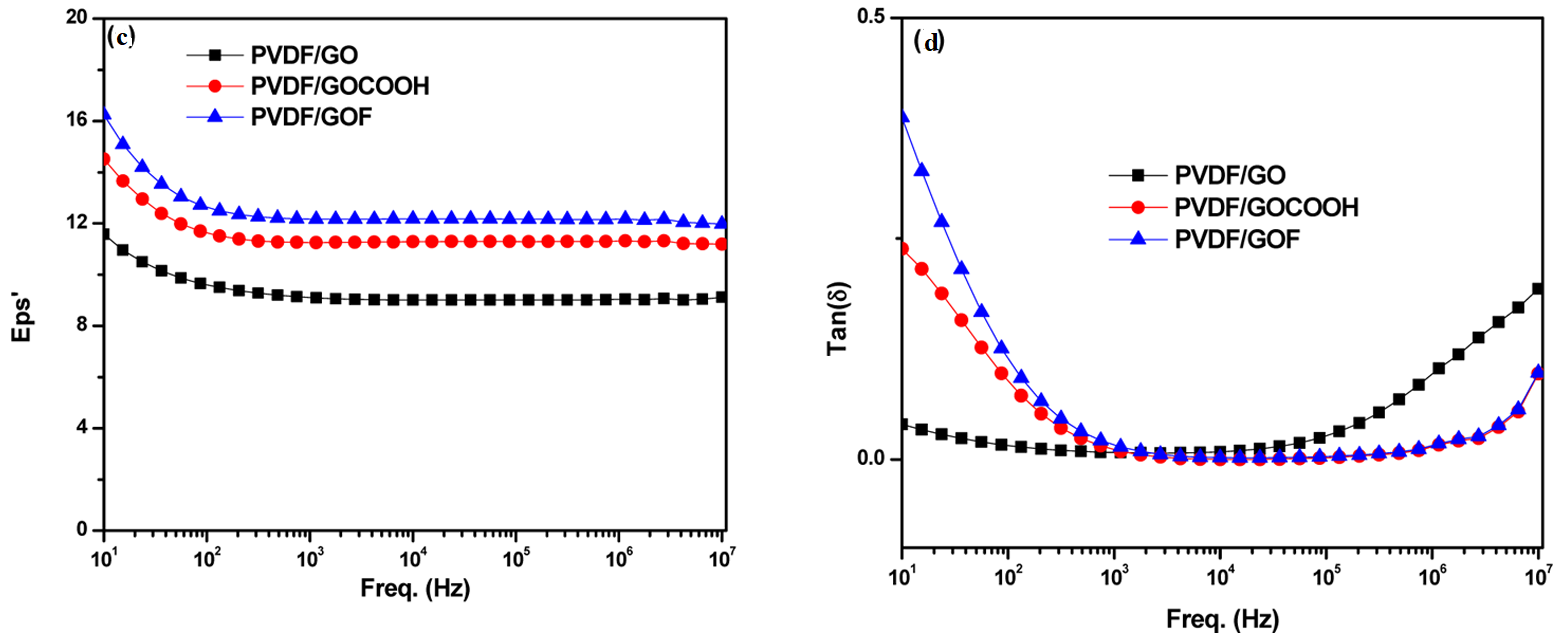


**Figure S2.** Piezoelectric force microscopy (PFM) amplitude vs dc voltage (voltage varying from −12 to 12 V) hysteresis loops for (**a**) PVDF/GOCOOH and (**b**) PVDF/GOF. Frequency dependence of (**c**) dielectric constant and (d) dielectric loss for PVDF/GO, PVDF/GOCOOH, and PVDF/GOF composites. (**e**) schematic representing the P−E loops for PVDF/GO, PVDF/GOCOOH, and PVDF/ GOF at room temperature. Reproduced with permission from (Gebrekrstos et al., 2018) Copyright 2018 American Chemical Society.

**Table S3**. Summary of studies on the improvement of various properties when adding GNMs in the polymer spinnable solution

| **GNMs/Polymer** | **Spinning parameters** | **Improved properties** | **Potential Applications** | **Remarks** | **Reference** |
| --- | --- | --- | --- | --- | --- |
| Polyacrylonitrile (PAN)/GO | (15 cm; 15 kV; 0.8 mL/h)  GO=0.4%wt | Mechanical strength (by 3-4 times)  The thermal stability  Hydrophilicity (by 50%) | Water treatment and battery performance | The content of GO influences its dispersion and thus may affect the fiber formation as well as the final performance and properties of ES fibers. | (Hou, Yun, & Byun, 2019) |
| GO/GR/Halloysite NT/PVDF | (10 cm; 16 kV; 2mL/h) | ﻿Piezoelectric and pyroelectric  The thermal stability (by 94%) Young’s modulus increased by 20 times | Wearable electronics and energy harvesting applications from body movements | The content of the nanofillers shows significant effect on piezoelectric responses due to enhancement of electroactive β‐phase. | (Abbasipour et al., 2019) |
| GO/PEO/PAN | (20 cm; 20 kV; 1 mL/h) | electrolyte uptake  ionic conductivity | The content of GO influences the fiber diameter and ionic conductivity | Homogenous distribution of GO fillers in the polymer matrix causes increase in the electrolyte uptake and electrical conductivity of the nanofibers. | (Abdollahi, Ehsani, Morshedian, Khonakdar, & Aram, 2019) |
| PCL/rGO | (10cm; 10, 15kV, 6mL/h) s | Mechanical behaviour Electrical conductivity Thermal stability | ﻿Human tissue repair | The evaluated properties were affected according to the amount of rGO used and the applied voltage | (Correa, Moncada, Gutiérrez, Vargas, & Zapata, 2019) |
| GO/PET | (12 cm; 10 kV; 0.1 mL/h) | Young modulus by 50% MPa.  The electroconductivity is improved | Improve cell attachment and proliferation | The GO, spinning parameters and concentration control the electroconductivity, mechanical properties and the uniformity of NF | (Ghasemi et al., 2019) |
| PU-GO-PDA | (20 cm; 20 kV; 0.2 mL/h) | Wettability, water absorption, and both cell attachment and proliferation and ALP expression rate which denotes osteogenic differentiation property | Bone regeneration of tissues | ﻿PU/GO was prepared by electrospinning and then PDA was coated by immersing PU/GO NF in dopamine hydrochloride solution under constant stirring (1.5 mg/L in 10 mM of Tris buffer pH = 8.5) at room temperature in a dark environment. After 24 h, the scaffold was washed with deionized water three times, and air dried. | (Ghorbani, Zamanian, & Aidun, 2019) |
| GO/ (Sulfonated PVA) | (17 cm; 15 to 18 kV; 300 to 800 ﻿µL/h) | Thermal and hydrothermal stability  Proton conductivity  Retention of humiditys | Ionic polyelectrolyte membrane | The combination of the sulfonation, the crosslinking, and the addition of GO enhanced the proton conductivity | (Gil-Castell et al., 2019) |
| PVA/rGO | (6-10 cm; 15-25 kV; 10-20 ﻿﻿µL/min) | Tensile] strength (∼5 MPa) and and the elastic modulus (∼1.5 GPa)  Thermal stability  The electrical conductivities of the nanocomposites. | Biosensors, sensors etc. | The increment of rGO (1% wt) improved PVA NF properties due to the strong interfacial interaction between rGO and PVA.  rGO dispersion in the PVA solution did not alter the crystal structure of PVA | (Gozutok et al., 2019) |
| Ag/rGO/Polyamide (PI) | (20 kV;  ﻿DMAc: THF = 3:2, wt:wt %  Stirring | ﻿The λ, Tg and THRI values of the (Ag/rGO)/PI nanocomposites were all increased with increasing the Ag/ rGO filler loading. | ﻿- | ﻿The aggregation of rGO can be effectively restricted by introducing Ag nanoparticles.  ﻿  - | (Guo et al., 2019) |
| ﻿PVDF-Pt-Pd/RGO-CeO2 | 15 cm; 12 kV; 1 mL/h | Increased thermal and catalytic properties | ﻿DMFC applications | Novel electrospinning of PVDF-Pt-Pd/RGO-CeO2 nanocomposites | (Hanifah et al., 2019) |
| GO-doped PVDF/CuO/Al | 15 cm; 0.07 mm/min | Heat of reaction and reaction efficiency of PVDF/CuO/Al nanocomposites  Strong anti-oxidation capability | - | Electrospinning and GO doping can improve the reaction efficiency due to the improvement of microstructure quality and nanocomposites performance. | (Lyu et al., 2019) |
| ﻿PU and PU/rGO‐Ag | 17 cm; 18 kV; 0.3 mL/h | Tensile strength  Electrical conductivity Cardiogenic differentiation  Potential  Wettability | Cardiac tissue engineering | ﻿Adding rGO‐Ag and concentration influence the fiber diameter and the final properties | (Nazari et al., 2019) |
| MnO2-GO | 10 cm; 15 kV; 0.5 mL/h | electrochemical  dielectric behaviors  higher charge mobility, diffusivity, and conductivity. | Future energy storage devices. | - | (Saha et al., 2019) |
| ﻿PCL/GO-Gelatin | 13 cm; 14 kV; 3 mL/h | Tensile stress and  Young’s modulus | Anti-tumor effect of classical therapies | ﻿The presence of 1 wt% graphene oxide increased mechanical strength of PCL/Gel | (Unal et al., 2019) |

**References**

Abbasipour, M., Khajavi, R., Yousefi, A. A., Yazdanshenas, M. E., & Razaghian, F. (2017). The piezoelectric response of electrospun PVDF nanofibers with graphene oxide, graphene, and halloysite nanofillers: a comparative study. *Journal of Materials Science: Materials in Electronics*, *28*(21), 15942–15952. https://doi.org/10.1007/s10854-017-7491-4

Abbasipour, M., Khajavi, R., Yousefi, A. A., Yazdanshenas, M. E., Razaghian, F., & Akbarzadeh, A. (2019). Improving piezoelectric and pyroelectric properties of electrospun PVDF nanofibers using nanofillers for energy harvesting application. *Polymers for Advanced Technologies*, *30*(2), 279–291. https://doi.org/10.1002/pat.4463

Abdali, H., & Ajji, A. (2017). Preparation of electrospun nanocomposite nanofibers of polyaniline/poly(methyl methacrylate) with amino-functionalized graphene. *Polymers*, *9*(9), 453. https://doi.org/10.3390/polym9090453

Abdollahi, S., Ehsani, M., Morshedian, J., Khonakdar, H. A., & Aram, E. (2019). Application of response surface methodology in assessing the effect of electrospinning parameters on the morphology of polyethylene oxide/polyacrylonitrile blend nanofibers containing graphene oxide. *Polymer Bulletin*, *76*(4), 1755–1773. https://doi.org/10.1007/s00289-018-2448-1

Aboamera, N. M., Mohamed, A., Salama, A., Osman, T. A., & Khattab, A. (2018). An effective removal of organic dyes using surface functionalized cellulose acetate/graphene oxide composite nanofibers. *Cellulose*, *25*(7), 4155–4166. https://doi.org/10.1007/s10570-018-1870-8

Abolhasani, M. M., Shirvanimoghaddam, K., & Naebe, M. (2017). PVDF/graphene composite nanofibers with enhanced piezoelectric performance for development of robust nanogenerators. *Composites Science and Technology*, *138*, 49–56. https://doi.org/10.1016/j.compscitech.2016.11.017

Ahmed, A., Jia, Y., Huang, Y., Khoso, N. A., Deb, H., Fan, Q., & Shao, J. (2019). Preparation of PVDF-TrFE based electrospun nanofibers decorated with PEDOT-CNT/rGO composites for piezo-electric pressure sensor. *Journal of Materials Science: Materials in Electronics*, (0123456789). https://doi.org/10.1007/s10854-019-01751-w

Andre, R. S., Mercante, L. A., Facure, M. H. M., Mattoso, L. H. C., & Correa, D. S. (2019). Enhanced and selective ammonia detection using In 2 O 3 /reduced graphene oxide hybrid nanofibers. *Applied Surface Science*, *473*, 133–140. https://doi.org/10.1016/j.apsusc.2018.12.101

Bahrami, S., Solouk, A., Mirzadeh, H., & Seifalian, A. M. (2019). Electroconductive polyurethane/graphene nanocomposite for biomedical applications. *Composites Part B: Engineering*, *168*(March), 421–431. https://doi.org/10.1016/j.compositesb.2019.03.044

Banitaba, S. N., Semnani, D., Heydari‐Soureshjani, E., Rezaei, B., & Ensafi, A. A. (2019). Nanofibrous poly(ethylene oxide)‐based structures incorporated with multi‐walled carbon nanotube and graphene oxide as all‐solid‐state electrolytes for lithium ion batteries. In *Polymer International* (Vol. 68). https://doi.org/10.1002/pi.5889

Choi, Y. I., Hwang, B. U., Meeseepong, M., Hanif, A., Ramasundaram, S., Trung, T. Q., & Lee, N. E. (2019). Stretchable and transparent nanofiber-networked electrodes based on nanocomposites of polyurethane/reduced graphene oxide/silver nanoparticles with high dispersion and fused junctions. *Nanoscale*, *11*(9), 4015–4024. https://doi.org/10.1039/c8nr10170a

Correa, E., Moncada, M. E., Gutiérrez, O. D., Vargas, C. A., & Zapata, V. H. (2019). Characterization of polycaprolactone/rGO nanocomposite scaffolds obtained by electrospinning. *Materials Science and Engineering C*, *103*(30), 109773. https://doi.org/10.1016/j.msec.2019.109773

Darzi, M. E., Golestaneh, S. I., Kamali, M., & Karimi, G. (2019). Thermal and electrical performance analysis of co-electrospun-electrosprayed PCM nanofiber composites in the presence of graphene and carbon fiber powder. *Renewable Energy*, *135*, 719–728. https://doi.org/10.1016/j.renene.2018.12.028

Davoodi, A. H., Mazinani, S., Sharif, F., & Ranaei-Siadat, S. O. (2018). GO nanosheets localization by morphological study on PLA-GO electrospun nanocomposite nanofibers. *Journal of Polymer Research*, *25*(9), 16–19. https://doi.org/10.1007/s10965-018-1589-0

Du, F., Sun, L., Huang, Z., Chen, Z., Xu, Z., Ruan, G., & Zhao, C. (2020). Electrospun reduced graphene oxide/TiO2/poly(acrylonitrile-co-maleic acid) composite nanofibers for efficient adsorption and photocatalytic removal of malachite green and leucomalachite green. *Chemosphere*, *239*, 124764. https://doi.org/10.1016/j.chemosphere.2019.124764

Gebrekrstos, A., Madras, G., & Bose, S. (2018). Piezoelectric Response in Electrospun Poly(vinylidene fluoride) Fibers Containing Fluoro-Doped Graphene Derivatives [Research-article]. *ACS Omega*, *3*(5), 5317–5326. https://doi.org/10.1021/acsomega.8b00237

Ghaderi, G., Tavanai, H., & Bazarganipour, M. (2019). Electrospun graphene oxide incorporated PAN nanofibers, before and after activation. *Materials Research Express*, *6*(10), 105047.

Ghasemi, A., Imani, R., Yousefzadeh, M., Bonakdar, S., Solouk, A., & Fakhrzadeh, H. (2019). Studying the Potential Application of Electrospun Polyethylene Terephthalate/Graphene Oxide Nanofibers as Electroconductive Cardiac Patch. *Macromolecular Materials and Engineering*, *304*(8), 1900187. https://doi.org/10.1002/mame.201900187

Ghorbani, F., Zamanian, A., & Aidun, A. (2019). Bioinspired polydopamine coating-assisted electrospun polyurethane-graphene oxide nanofibers for bone tissue engineering application. *Journal of Applied Polymer Science*, *136*(24), 1–9. https://doi.org/10.1002/app.47656

Gil-Castell, O., Galindo-Alfaro, D., Sánchez-Ballester, S., Teruel-Juanes, R., Badia, J. D., & Ribes-Greus, A. (2019). Crosslinked sulfonated poly(Vinyl alcohol)/graphene oxide electrospun nanofibers as polyelectrolytes. *Nanomaterials*, *9*(3), 1–20. https://doi.org/10.3390/nano9030397

Gozutok, M., Sadhu, V., & Sasmazel, H. T. (2019). Development of Poly(vinyl alcohol) (PVA)/Reduced Graphene Oxide (rGO) Electrospun Mats. *Journal of Nanoscience and Nanotechnology*, *19*(7), 4292–4298. https://doi.org/10.1166/jnn.2019.16290

Gu, X., Li, Y., Cao, R., Liu, S., Fu, C., Feng, S., … Wang, Y. (2019). Novel electrospun poly(lactic acid)/poly(butylene carbonate)/graphene oxide nanofiber membranes for antibacterial applications. *AIP Advances*, *9*(6). https://doi.org/10.1063/1.5100109

Guo, Y., Yang, X., Ruan, K., Kong, J., Dong, M., Zhang, J., … Guo, Z. (2019). Reduced Graphene Oxide Heterostructured Silver Nanoparticles Significantly Enhanced Thermal Conductivities in Hot-Pressed Electrospun Polyimide Nanocomposites [Research-article]. *ACS Applied Materials & Interfaces*, *11*(28), 25465–25473. https://doi.org/10.1021/acsami.9b10161

Hanifah, M. F. R., Jaafar, J., Othman, M. H. D., Ismail, A. F., Rahman, M. A., Yusof, N., & Aziz, F. (2019). Electro-spun of novel PVDF-Pt-Pd/RGO-CeO2 composite nanofibers as the high potential of robust anode catalyst in direct methanol fuel cell: Fabrication and characterization. *Inorganic Chemistry Communications*, *107*(July), 107487. https://doi.org/10.1016/j.inoche.2019.107487

Heidari, M., Bahrami, H., & Ranjbar-Mohammadi, M. (2017). Fabrication, optimization and characterization of electrospun poly(caprolactone)/gelatin/graphene nanofibrous mats. *Materials Science and Engineering C*, *78*, 218–229. https://doi.org/10.1016/j.msec.2017.04.095

Hou, J., Yun, J., & Byun, H. (2019). Fabrication and Characterization of Modified Graphene Oxide/PAN Hybrid Nanofiber Membrane. *Membranes*, *9*(9), 122. https://doi.org/10.3390/membranes9090122

Huang, C. L., Wu, H. H., Jeng, Y. C., & Liang, W. Z. (2019). Electrospun graphene nanosheet-filled poly(trimethylene terephthalate) composite fibers: Effects of the graphene nanosheet content on morphologies, electrical conductivity, crystallization behavior, and mechanical properties. *Polymers*, *11*(1). https://doi.org/10.3390/polym11010164

Jahan Biglari, M., Semnani Rahbar, R., Shabanian, M., & Khonakdar, H. A. (2018). Novel composite nanofibers based on polyamide 66/graphene oxide- grafted aliphatic- aromatic polyamide: preparation and characterization. *Polymer - Plastics Technology and Engineering*, *00*(00), 1–10. https://doi.org/10.1080/03602559.2018.1542712

Javed, K., Krumme, A., Viirsalu, M., Krasnou, I., Plamus, T., Vassiljeva, V., … Chen, C. M. (2018). A method for producing conductive graphene biopolymer nanofibrous fabrics by exploitation of an ionic liquid dispersant in electrospinning. *Carbon*, *140*, 148–156. https://doi.org/10.1016/j.carbon.2018.08.034

Li, F., Peng, H., Xia, D., Yang, J., Yang, K., Yin, F., & Yuan, W. (2019). Highly Sensitive, Selective, and Flexible NO 2 Chemiresistors Based on Multilevel Structured Three-Dimensional Reduced Graphene Oxide Fiber Scaffold Modified with Aminoanthroquinone Moieties and Ag Nanoparticles [Research-article]. *ACS Applied Materials and Interfaces*, *11*(9), 9309–9316. https://doi.org/10.1021/acsami.8b20462

Li, H., Shi, W., Zeng, X., Huang, S., Zhang, H., & Qin, X. (2020). Improved desalination properties of hydrophobic GO-incorporated PVDF electrospun nanofibrous composites for vacuum membrane distillation. *Separation and Purification Technology*, *230*(February 2019), 115889. https://doi.org/10.1016/j.seppur.2019.115889

Lyu, J. Y., Chen, S., He, W., Zhang, X. X., Tang, D. yun, Liu, P. J., & Yan, Q. L. (2019). Fabrication of high-performance graphene oxide doped PVDF/CuO/Al nanocomposites via electrospinning. *Chemical Engineering Journal*, 129–137. https://doi.org/10.1016/j.cej.2019.02.170

Maccaferri, E., Mazzocchetti, L., Benelli, T., Zucchelli, A., & Giorgini, L. (2019). Morphology, thermal, mechanical properties and ageing of nylon 6,6/graphene nanofibers as Nano2 materials. *Composites Part B: Engineering*, *166*(November 2018), 120–129. https://doi.org/10.1016/j.compositesb.2018.11.096

Nazari, H., Azadi, S., Hatamie, S., Zomorrod, M. S., Ashtari, K., Soleimani, M., & Hosseinzadeh, S. (2019). Fabrication of graphene-silver/polyurethane nanofibrous scaffolds for cardiac tissue engineering. *Polymers for Advanced Technologies*, *30*(8), 2086–2099. https://doi.org/10.1002/pat.4641

Pan, S., Qi, Z., Li, Q., Ma, Y., Fu, C., Zheng, S., … Yang, X. (2019). Graphene oxide-PLGA hybrid nanofibres for the local delivery of IGF-1 and BDNF in spinal cord repair. *Artificial Cells, Nanomedicine and Biotechnology*, *47*(1), 651–664. https://doi.org/10.1080/21691401.2019.1575843

Parandeh, S., Kharaziha, M., & Karimzadeh, F. (2019). An eco-friendly triboelectric hybrid nanogenerators based on graphene oxide incorporated polycaprolactone fibers and cellulose paper. *Nano Energy*, *59*, 412–421. https://doi.org/10.1016/j.nanoen.2019.02.058

Pavinatto, A., Mercante, L. A., Facure, M. H. M., Pena, R. B., Sanfelice, R. C., Mattoso, L. H. C., & Correa, D. S. (2018). Ultrasensitive biosensor based on polyvinylpyrrolidone/chitosan/reduced graphene oxide electrospun nanofibers for 17α – Ethinylestradiol electrochemical detection. *Applied Surface Science*, *458*(June), 431–437. https://doi.org/10.1016/j.apsusc.2018.07.035

Ren, J., Woo, Y. C., Yao, M., Lim, S., Tijing, L. D., & Shon, H. K. (2019). Nanoscale zero-valent iron (nZVI) immobilization onto graphene oxide (GO)-incorporated electrospun polyvinylidene fluoride (PVDF) nanofiber membrane for groundwater remediation via gravity-driven membrane filtration. *Science of the Total Environment*, *688*, 787–796. https://doi.org/10.1016/j.scitotenv.2019.05.393

Ruan, K., Guo, Y., Tang, Y., Zhang, Y., Zhang, J., He, M., … Gu, J. (2018). Improved thermal conductivities in polystyrene nanocomposites by incorporating thermal reduced graphene oxide via electrospinning-hot press technique. *Composites Communications*, *10*(June), 68–72. https://doi.org/10.1016/j.coco.2018.07.003

Ruiz, V., Pérez-Marquez, A., Maudes, J., Grande, H. J., & Murillo, N. (2018). Enhanced photostability and sensing performance of graphene quantum dots encapsulated in electrospun polyacrylonitrile nanofibrous filtering membranes. *Sensors and Actuators, B: Chemical*, *262*, 902–912. https://doi.org/10.1016/j.snb.2018.02.081

Saha, S., Maji, P., Pethsangave, D. A., Roy, A., Ray, A., Some, S., & Das, S. (2019). Effect of morphological ordering on the electrochemical performance of MnO2-Graphene oxide composite. *Electrochimica Acta*, *317*, 199–210. https://doi.org/10.1016/j.electacta.2019.05.148

Samani, D. A., Doostmohammadi, A., Nilforoushan, M. R., & Nazari, H. (2019). Electrospun Polycaprolactone/Graphene/Baghdadite Composite Nanofibres with Improved Mechanical and Biological Properties. *Fibers and Polymers*, *20*(5), 982–990. https://doi.org/10.1007/s12221-019-1161-5

Silvestri, D., Mikšíček, J., Wacławek, S., Torres-Mendieta, R., Padil, V. V. T., & Černík, M. (2019). Production of electrospun nanofibers based on graphene oxide/gum Arabic. *International Journal of Biological Macromolecules*, *124*, 396–402. https://doi.org/10.1016/j.ijbiomac.2018.11.243

Stone, H., Lin, S., & Mequanint, K. (2019). Preparation and characterization of electrospun rGO-poly(ester amide) conductive scaffolds. *Materials Science and Engineering C*, *98*(December 2018), 324–332. https://doi.org/10.1016/j.msec.2018.12.122

Tambakoozadeh, N., Youssefi, M., & Semnani, D. (2019). A composite polyaniline/graphene–coated polyamide6 nanofiber mat for electrochemical applications. *Polymers for Advanced Technologies*, (March), 1–8. https://doi.org/10.1002/pat.4714

Unal, S., Arslan, S., Gokce, T., Atasoy, B. M., Karademir, B., Oktar, F. N., & Gunduz, O. (2019). Design and characterization of polycaprolactone-gelatin-graphene oxide scaffolds for drug influence on glioblastoma cells. *European Polymer Journal*, *115*(March), 157–165. https://doi.org/10.1016/j.eurpolymj.2019.03.027

Wang, X., Gao, Y., Li, X., Xu, Y., Jiang, J., Hou, J., … Turng, L. S. (2017). Selective localization of graphene oxide in electrospun polylactic acid/poly(ε-caprolactone) blended nanofibers. *Polymer Testing*, *59*, 396–403. https://doi.org/10.1016/j.polymertesting.2017.02.022

Weng, R., Sun, L., Jiang, L., Li, N., Ruan, G., Li, J., & Du, F. (2019). Electrospun Graphene Oxide–Doped Nanofiber-Based Solid Phase Extraction Followed by High-Performance Liquid Chromatography for the Determination of Tetracycline Antibiotic Residues in Food Samples. *Food Analytical Methods*, *12*(7), 1594–1603. https://doi.org/10.1007/s12161-019-01495-7
